# Supplementary material for: The antimicrobial potential of cannabidiol
Source: Commun Biol. 2021 Jan 19;4:7. doi: 10.1038/s42003-020-01530-y (PMC7815910; doi:10.1038/s42003-020-01530-y)
Supplement: Supplementary file 2 — Supplementary Information [file 42003_2020_1530_MOESM2_ESM.pdf]

Supplementary Material

The antimicrobial potential of cannabidiol

Mark A.T. Blaskovich[1]*, Angela M. Kavanagh[1], Alysha G. Elliott[1], Bing Zhang[1], Soumya Ramu[1], Maite Amado[1], Gabrielle J. Lowe[1], Alexandra O. Hinton[1], Do Minh Thu Pham[1], Johannes Zuegg[1], Neil Beare[2], Diana Quach[3], Marc D. Sharp[3], Joe Pogliano[3,4], Ashleigh P. Rogers[5], Dena Lyras[5], Lendl Tan[6], Nicholas P. West,[6] David W. Crawford[7], Marnie Peterson[7], Matthew Callahan[8], and Michael Thurn[8]

[1] Centre for Superbug Solutions, Institute for Molecular Bioscience, The University of Queensland, St. Lucia, Queensland 4072, Australia
[2] BDG Synthesis, Wellington 5045, New Zealand
[3] Linnaeus Bioscience Inc., 3210 Merryfield Row, San Diego, CA 92121, USA
[4] Division of Biological Sciences, University of California, San Diego, CA 92093, USA
[5] Infection and Immunity Program, Monash Biomedicine Discovery Institute and Department of Microbiology, Monash University, Clayton, Victoria 3800, Australia
[6] School of Chemistry and Molecular Biosciences, The University of Queensland, St Lucia, Queensland 4072, Australia
[7] Extherid Biosciences, 3545 S Park Dr, Jackson, WY83001 USA
[8] Botanix Pharmaceuticals Ltd, Level 1, 50 Angove Street North Perth, Western Australia 6005, Australia

Supplementary figures

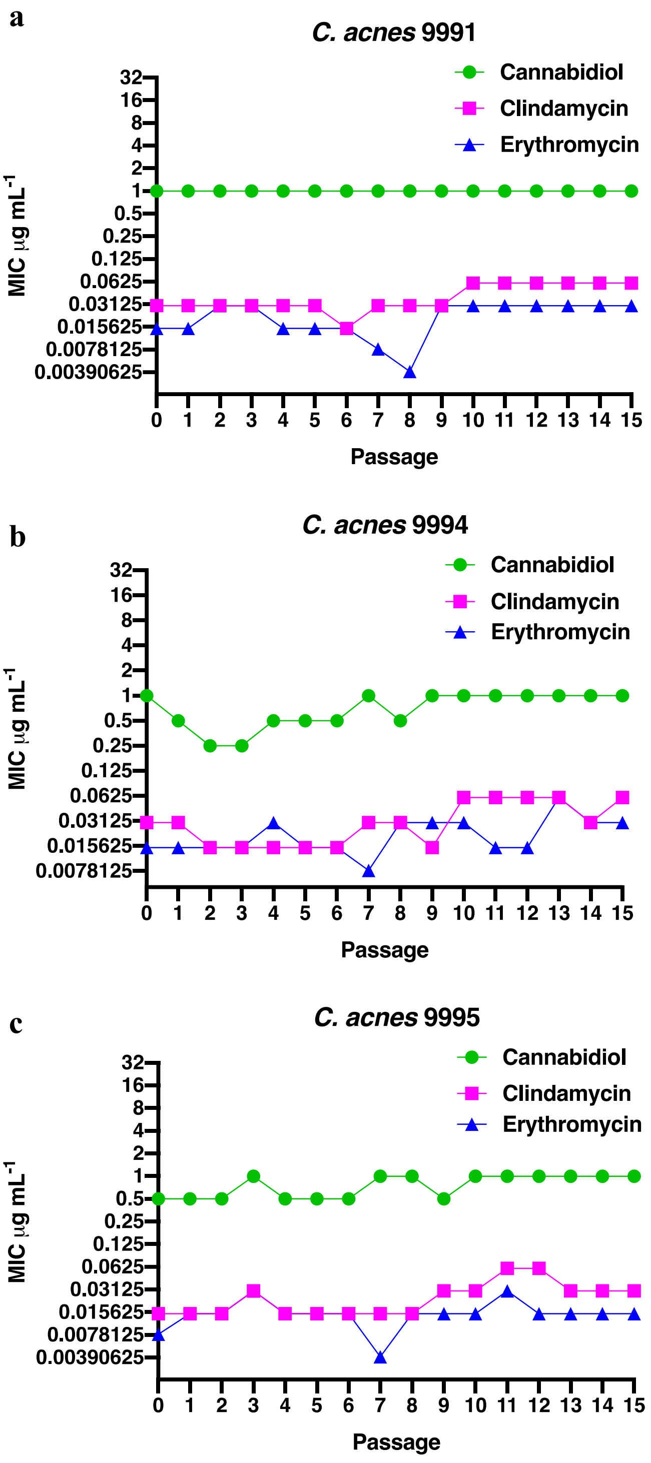

Supplementary Fig. 1: Broth microdilution serial passage resistance induction studies. a, b, c Induction studies performed for three *C. acnes* strains over 15 passages, with each passage requiring anaerobic incubation for 46–48 h at 35–37 °C; n = 1.

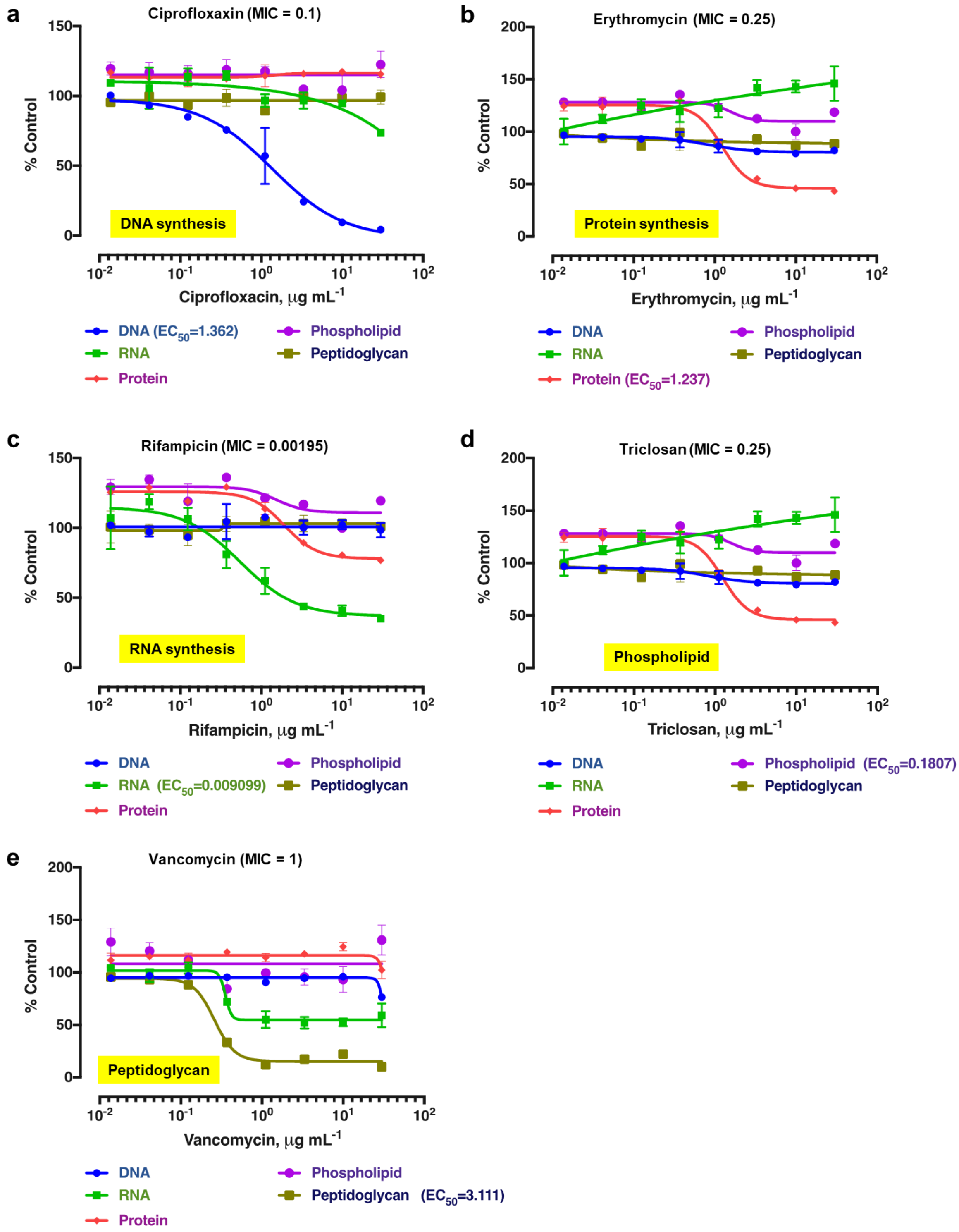

Supplementary Fig. 2: Macromolecular synthesis.

Control antibiotics tested in macromolecular synthesis assay in *S. aureus* RN42200 showing inhibition of radiolabelled substrate uptake in (**a**) DNA ([2-^{14}C]-thymidine), (**b**) protein (L-[4,5-^{3}H]-leucine), (**c**) RNA ([5,6-^{3}H]-uracil), (**d**) phospholipid ([2-^{3}H]-glycerol) and (**e**) peptidoglycan ([^{14}C(U)]-glycine) synthesis pathways after 35 min incubation. MIC values are displayed as µg mL^{-1}. Data are mean ± SD for n=2 biologically independent samples.

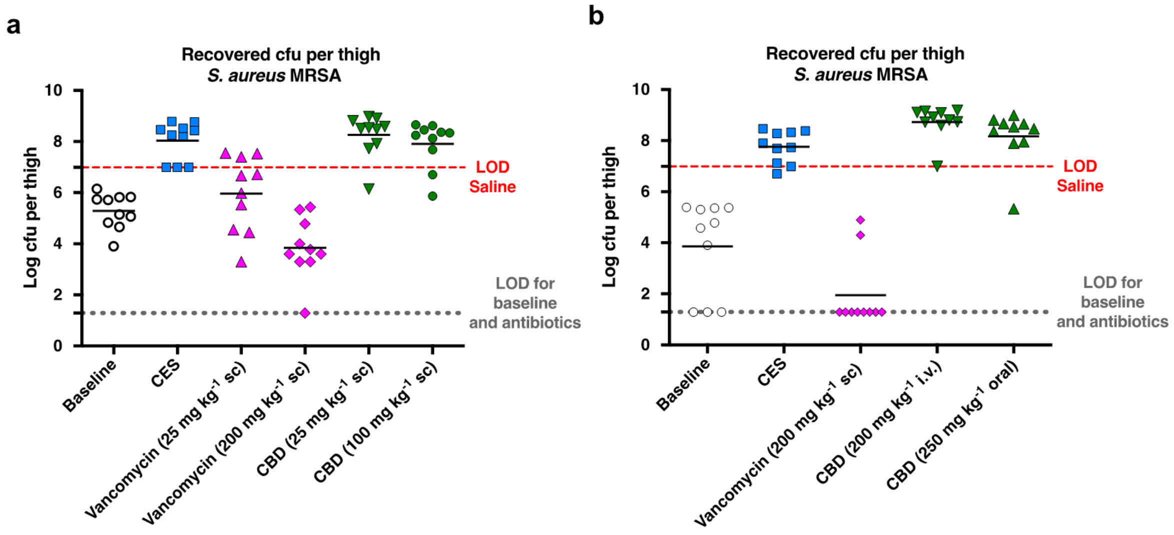

Supplementary Fig. 3: MRSA murine thigh infection model.

Immunocompromised mice (n=5 group) were inoculated in both thighs with 1×10^6 CFU of *S. aureus* ATCC 43300 (MRSA) bacteria. One group was sacrificed 2 h after infection for a baseline measurement, at the same time as treatment was initiated with (**a**) subcutaneous doses of saline, vancomycin (25 and 200 mg kg^{-1}) and CBD (25 and 100 mg kg^{-1}), or (**b**) subcutaneous saline and vancomycin (200 mg kg^{-1}), oral CBD (250 mg kg^{-1}) and intraperitoneal (ip) CBD (200 mg kg^{-1}). Animals were sacrificed 24 h after dosing, thighs were excised and homogenised, then diluted and plated to determine CFU/thigh levels. Data are n = 5 animals, with n = 2 biological samples per mouse (both thighs). Errors are mean ± SEM. CES, saline negative control; LOD, level of detection.

Supplementary Table 1. Minimum inhibitory concentrations (MICs) of CBD (µg mL^{-1}) against Gram-positive bacteria.

Species	Strain	Vanco-mycin	Dapto-mycin	Trimeth-oprim	Mupir-ocin	Clinda-mycin	CBD
Staphylococcus aureus[1]	ATCC 25923 MSSA	1–2	1–2	2–4	0.25–0.5	0.06–0.25	1–2
Staphylococcus aureus[1]	ATCC 29213 MSSA	0.5–1	0.5–2	2–4	0.25–0.5	0.06–0.25	1–2
Staphylococcus aureus[1]	ATCC 43300 MRSA	0.5–1	0.5–1	2	0.125–0.5	>64	1–2
Staphylococcus aureus[1]	ATCC 33591 MRSA	0.5–2	1–2	1–2	0.125–0.5	>64	1–2
Staphylococcus aureus[1]	Clinical isolate MRSA Daptomycin resistant	1–2	2-4	>64	0.125–0.25	>64	2–8
Staphylococcus aureus[1]	ATCC 700699, NRS-1 MRSA VISA	2–4	4-8	2-4	0.125–0.5	64	1–4
Staphylococcus aureus[1]	VRS1 VRSA	>64	1–4	>64	32–64	>64	1–2
Staphylococcus capitis[1]	ATCC 27840	0.5–1	1	2–4	0.125–0.25	0.06–0.125	1–2
Staphylococcus epidermidis[1]	ATCC 12228	1–2	1-8	1–4	0.25–0.5	0.06–0.25	1–2
Staphylococcus epidermidis[1]	ATCC 14990	1–2	1	0.25-0.5	0.125–0.25	0.03–0.125	1–2
Staphylococcus epidermidis[1]	NRS 60 VISE	2–4	1–4	>64	0.125–0.5	0.06–0.125	4–8
Staphylococcuss saprophyticus[1]	ATCC 15305	1–2	1	2–1	≤0.03	0.125–0.25	1–2
Staphylococcus warneri[1]	ATCC 27836	0.5–2	1	1–2	0.5	0.06–0.125	2–4
Staphylococcus warneri[1]	Clinical isolate	0.5–1	1	0.25–0.5	0.25–0.5	0.06–0.125	2–4
Streptococcus canis[1]	ATCC 43496	≤0.03–0.125	≤0.03–0.125	0.25–1	≤0.03	0.25	0.5–1
Streptococcus pneumoniae[11]	ATCC 33400	0.5–1	1-4	0.5–2	0.25–0.5	0.06–2	1–2
Streptococcus pneumoniae[1]	ATCC 700677 MDR	1	1–4	1–2	0.25–0.5	>64	1–4
Streptococcus intermedius[1]	ATCC 43496	0.5–1	0.5	4–8	0.06–0.125	0.125	1–2
Streptococcus pyogenes[1]	ATCC 12344	0.25–0.5	0.125–0.25	2–4	≤0.03	0.125–0.25	1
Streptococcus pyogenes[1]	ATCC 49399	0.125–0.25	0.06	≤0.03	≤0.03	0.125	0.5
Streptococcus pyogenes[1]	ATCC 19615	0.25	0.06	0.5	≤0.03	0.06–0.125	1
Streptococcus pyogenes[1]	ATCC BAA-1412	0.25	0.06	≤0.03–0.06	32–>64	≤0.03–0.06	1
Streptococcus pyogenes[1]	ATCC BAA-1414	0.125–0.25	≤0.03–0.06	≤0.06–0.125	≤0.03	≤0.03–0.06	1
Bacillus cereus[1]	ATCC 11778	0.125–1	1–4	0.25-1	0.25–0.5	0.5–2	1–2
Bacillus megaterium[1]	ATCC 13632	0.06–0.125	1–2	2	32->64	8–32	1–2
Bacillus subtilis[1]	ATCC 6633	0.06–0.5	0.5–2	0.125–0.25	0.25–0.5	0.5–2	1–2
Corynebacterium minutissimum[1]	ATCC23347	0.125–0.25	0.06–0.125	>64	1	>64	1
Corynebacterium minutissimum[1]	ATCC23348	0.125–0.25	0.06–0.125	>64	1	>64	1-2
Enterococcus faecium[1]	ATCC 35667	0.25–0.5	4–8	≤0.03–0.06	1	≤0.03–0.06	0.5–1
Enterococcus faecium	ATCC700221 VRE	>64	8-16	8-16	1-2	>64	1-2
Enterococcus faecalis[1]	ATCC 29212	1–2	4–8	0.25–0.5	64	16-32	2
Enterococcus faecalis	clinical isolate VRE	32	16	0.5-1	>64	>64	2-4
Enterococcus faecalis	clinical isolate VRE	>64	8-16	>64	>64	>64	2-4
Kocuria rosea[1]	ATCC 31251	1–2	1	1-2	0.06–0.125	4–16	1–2
	**Strain**	**Vanco-mycin**		**Levo-floxacin**	**Mero-penem**	**Genta-micin**	**CBD**
Staphylococcus aureus[2]	ATCC 29213	1		0.25	0.06	0.25	2
Staphylococcus aureus[2]	ATCC 25923	1		0.25	0.12	0.25	2
Staphylococcus aureus[2]	ATCC 43300 MRSA	1		0.25	8	32	1
Staphylococcus aureus[2]	MMX 4665 VISA, DaptoNS	4		>32	32	32	1
Staphylococcus epidermidis[2]	MMX 5388 MSSE	2		0.25	≤0.03	≤0.03	1
Staphylococcus epidermidis[2]	MMX 5139 MRSE	0.5		>32	0.12	≤0.03	1
Staphylococcus haemolyticus[2]	MMX 7665	0.5		0.25	0.12	≤0.03	1
Enterococcus faecalis[2]	ATCC 29212	2		0.5	4	8	2
Enterococcus faecalis[2]	MMX 8958	0.5		1	4	>32	2
Enterococcus faecalis[2]	MMX 486 vanA VRE	>32		>32	4	>32	2
Enterococcus faecalis[2]	MMX 481 vanB VRE	>32		16	4	>32	1
Enterococcus faecium[2]	ATCC 19434	0.25		4	8	4	0.5
Enterococcus faecium[2]	MMX 485 vanA VRE	>32		2	>32	>32	1
Enterococcus gallinarum[2]	ATCC 700425	4		1	32	8	1
Streptococcus pneumoniae[2]	ATCC 49619 PISP	0.12		0.5	≤0.03	8	1
Streptococcus pneumoniae[2]	MMX 7808 PRSP	0.12		1	0.25	8	8
Streptococcus pneumoniae[2]	MMX 5440 MDR	0.25		1	0.5	4	16
Streptococcus pyogenes[2]	ATCC 19615	0.25		0.5	≤0.03	4	16
Streptococcus pyogenes[2]	MMX 3820 ERY^{R}	0.25		0.25	≤0.03	8	16

Streptococcus agalactiae[2]	MMX 4088	0.5		1	≤0.03	32	16
Streptococcus agalactiae[2]	MMX 4115 MLS	0.5		1	≤0.03	16	16
Group C *Streptococcus*[2]	MMX 5125 ERY^{R}	0.5		0.5	≤0.03	8	1
Group G *Streptococcus*[2]	MMX 5121	0.25		0.5	≤0.03	8	16
Streptococcus constellatus[2]	MMX 5677	0.25		1	≤0.03	2	8
Streptococcus oralis[2]	MMX 5821 ERY^{R}	0.25		1	≤0.03	4	16
Streptococcus mutans[2]	ATCC 25175	0.5		1	≤0.03	1	16
Corynebacterium jeikeium[2]	ATCC 43734	0.5		0.25	1	>32	16
Corynebacterium striatum[2]	ATCC 6940	0.25		0.25	0.12	≤0.03	16
Listeria monocytogenes[2]	ATCC 19115	1		1	0.12	0.25	32

Gram-Positive: Anaerobic Conditions

Species	Strain	Vanco-mycin	Erythro-mycin	Tetra-cycline	Mupir-ocin	Clinda-mycin	CBD
Staphylococcus aureus[1]	ATCC 43300 MRSA	0.5-1	>32	0.06-0.25	0.03-0.06	>32	1-2
Propionibacterium acnes[1]	ATCC 6919	0.25	0.125-0.25	0.125-0.5	>32	0.125	1-2
Acidipropionibacterium acidipropionici[1]	ATCC 25562	0.125-0.25	2-4	0.125-0.5	>32	0.125	0.5
Cutibacterium granulosum[1]	ATCC 25564	0.25	0.125	0.125-0.25	>32	0.03-0.125	2–4
	**Strain**				**Metro-nidazole**	**Clinda-mycin**	**CBD**
Clostridium difficile[2]	ATCC 700057				0.12	1	32
Clostridium perfringens[2]	MMX 9784				2	1	32
Gemella morbillorum[2]	MMX 3433				>32	16	16
Actinomyces naeslundii[2]	MMX 627				32	0.5	8
Eggerthella lenta[2]	ATCC 43055				0.25	0.12	32
Lactobacillus acidophilus[2]	ATCC 4356				>32	1	32
Bifidobacterium longum[2]	MMX 9739				>32	≤0.03	16
Clostridium difficile Canadian hypervirulent ribotype 027 human[3]			M7404				2–4
Clostridium difficile U.K. hypervirulent ribotype 027 human strain[3]			R20291				2–4
Clostridium difficile variant toxin B ribotype 017 human (no Toxin A)[3]			1470				2–4
Clostridium difficile ribotype RT078, US animal strain, infects humans. Produces third toxin, CDT[3]			JGS6133				2-4
Clostridium difficile ribotype RT237 Australian animal strain, infects humans. Uncommon toxin profile of Toxin A negative, Toxin B positive, CDT (binary toxin) positive[3]			AI35				2-4
	**Strain**			**CBD + 5%LHB**	**Erythro-mycin**	**Clinda-mycin**	**CBD**
Cutibacterium acnes[2]	MMX 9991			8	0.03	0.12	1
Cutibacterium acnes[2]	MMX 9994			16	4	0.03	1
Cutibacterium acnes[2]	MMX 9995			16	0.03	0.06	2
Cutibacterium acnes[2]	MMX 9997			16	0.03	0.03	2
Cutibacterium acnes[2]	MMX 9999			16	0.03	0.5	1
Bacteroides fragilis[2]	ATCC 25285			32	4	0.5	2
Bacteroides thetaiotaomicron[2]	ATCC 29741			64	8	4	4

[1]MIC assays at UQ (n = ≥ 4, ≥ 2 independent duplicates)

[2]MIC assays at Micromyx (n = 3, triplicate independent inocula)

[3]MIC assays at Monash (n = 2)

ERY^{R}, erythromycin resistant; MDR, multi-drug resistant; MLS, macrolide-lincosamide-streptoramin resistant; MRSA, methicillin-resistant *S. aureus*; MRSE, methicillin-resistant *S. epidermidis*; MSSA, methicillin-susceptible *S. aureus*; MSSE, methicillin-susceptible *S. epidermidis*; PISP, penicillin-intermediate *S. pneumoniae*; PRSP, penicillin-resistant *S. pneumoniae*; VRE, vancomycin-resistant enterococci; VSE, vancomycin-susceptible enterococci;

Supplementary Table 2. ***S. aureus*** **MIC90** (ATCC and Australian clinical isolates, n = 4)

Strain				MIC (µg mL^{-1}) *(individual values, and average)*																								
				**Vancomycin**					**Daptomycin**					**Mupirocin**					**Clindamycin**					**CBD**				
MSSA	ATCC 25923	Control		2	1	1	8	2	2	2	2	2	2	0.5	0.5	0.5	1	0.5	0.06	0.06	0.06	0.125	0.06	2	2	1	1	2
MSSA	404556145	Clinical Isolate		2	1	1		1	4	2	2		2	0.5	0.5	0.5		0.5	64	64	64		64	2	2	2		2
MSSA	405575036	Clinical Isolate		1	1	1	1	1	2	2	1	2	2	0.25	0.25	0.5	0.5	0.5	0.5	0.125	0.125	0.125	0.125	2	2	1	1	2
MSSA	406626061	Clinical Isolate		0.5	0.5			0.5	2	2			2	0.25	0.5			0.5	0.125	0.06			0.125	2	2			2
MSSA	422940878	Clinical Isolate		1	1	2	1	1	2	2	2		2	0.5	0.5	0.25	0.25	0.5	0.125	0.125	0.125	0.125	0.125	2	2	1	1	2
MSSA	405573757	Clinical Isolate		1	1	1	1	1	2	2	2	2	2	0.5	0.5	0.25	0.25	0.5	0.125	0.125	0.06	0.06	0.125	2	2	2	2	2
MSSA	ATCC 29213	Control		1	1	1	1	1	2	2	2	1	2	0.25	0.25	0.25	0.5	0.25	0.06	0.06	0.06	0.125	0.06	2	2	1	1	2
MSSA	M30538	Clinical Isolate	Trachey swab	1	1	1	1	1	2	1	1	1	1	0.5	0.5	0.5	0.5	0.5	0.125	0.06	0.125	0.125	0.125	2	2	1	1	2
MSSA	M31394	Clinical Isolate	Finger	1	1	1	1	1	1	1	2	2	1	0.25	0.25	0.5	0.5	0.5	0.03	0.03	0.125	0.125	0.125	2	2	1	1	2
MSSA	M31634	Clinical Isolate	Toe	1	0.5	1	1	1	1	1	1	1	1	0.25	0.25	0.5	0.25	0.25	0.03	0.06	0.06	0.125	0.06	1	1	1	1	1
MSSA	M31907	Clinical Isolate	Abdo fluid	1	1	1	1	1	1	1	2	2	2	0.25	0.25	0.25	0.25	0.25	0.06	0.125	0.06	0.05	0.06	2	2	2	1	2
MSSA	M32158	Clinical Isolate	Pressure Area	1	1	1	1	1	1	1	2	2	2	0.25	0.25	0.5	0.25	0.25	0.03	0.03	0.06	0.125	0.125	2	2	2	2	2
MSSA	M32158	Clinical Isolate	Toe	0.5	1	1	1	1	2	1	2	2	2	0.5	0.5	1	0.5	0.5	0.125	0.125	0.06		0.125	2	2	1	1	2
MSSA	M34027	Clinical Isolate	Ankle	0.5	0.5	1	1	1	2	1	4	4	4	0.25	0.25	0.25	0.25	0.25	0.125	0.125	0.125	0.125	0.125	2	2	1	1	2
MSSA	M34575	Clinical Isolate	Wound	1	1	1	1	1	2	2	2	2	2	0.25	0.25	0.5	0.5	0.5	0.03	0.03	0.125	0.06	0.125	2	2	1	1	2
MSSA	M34591	Clinical Isolate	Neck	1	1	1	1	1	1	2	2	2	2	0.5	0.5	0.5	0.25	0.5	0.125	0.125	0.06	0.125	0.125	2	2	1	1	2
MSSA	M34593	Clinical Isolate	Wound	1	1	1	1	1	2	2	2	2	2	0.5	0.5	0.5	0.5	0.5	0.125	0.125	125	0.125	0.125	2	2	1	1	2
MSSA	M35252	Clinical Isolate	ETA	1	1	1	2	1	1	1	1	2	1	0.5	0.5	0.5	0.25	0.5	0.25	0.25	0.125		0.25	2	2	1	1	2
MSSA	M35254	Clinical Isolate	ETA	0.5	1	1	1	1	1	1	1	1	1	0.5	0.5	0.5	0.25	0.5	0.125	0.03	0.06	0.06	0.06	2	2	2	1	2
MSSA	M35255	Clinical Isolate	ETA	1	1			1	1	1			1	0.5	0.25			0.5	0.06	0.03			0.06	2	2			2
MSSA	M35264	Clinical Isolate	Arm	1	1	1		1	2	2	2		2	0.25	0.25	0.5		0.25	0.125	0.03	0.06		0.06	2	2	2		2
MSSA	M35268	Clinical Isolate	Trachey swab	1	1	1		1	2	2	2		2	0.5	0.25	2		0.5	0.125	0.125	0.5		0.125	2	2	1		2
MSSA	M35491	Clinical Isolate	Fluid Neck	1	1	1	1	1	2	2	1	2	2	0.5	0.5	0.5	0.5	0.5	0.125	0.06	0.125	0.125	0.125	2	2	2	2	2
MSSA	M35953	Clinical Isolate	Abcess	1	1	1	1	1	1	1	1	1	1	0.5	1	0.5	0.5	0.5	0.06	0.125	0.03	0.03	0.125	2	2	1	1	2
MSSA	M36523	Clinical Isolate	Urine	1	1	2	1	1	2	2	2	2	2	0.125	0.125	0.06	0.06	0.125	0.06	0.06	0.03	0.03	0.06	1	1	0.5	1	1
MSSA	M37410	Clinical Isolate	Abcess	1	1	1	1	1	1	1	2	2	2	64	64	64	64	64	64	64	64	64	64	2	2	1	1	2
MRSA	414149225	Clinical Isolate		1	1	1	0.25	1	2	2	0.5	0.25	2	2	0.5	0.125	0.25	2	0.125	0.125	0.06	0.125	0.125	2	2	1	1	2
MRSA	405574456	Clinical Isolate; Resistant		2	1	2	1	2	2	4	2	2	2	0.5	0.5	0.5	0.5	0.5	64	64	64	64	64	2	2	1	1	2
MRSA	ATCC 43300	Resistant		1	1	1	1	1	2	2	1	1	2	0.5	0.5	0.5	0.5	0.5	64	64	64	64	64	2	2	1	1	2
MRSA	ATCC 33591	Resistant		2	2	1	1	2	2	2	2	2	2	0.5	0.5	0.5	0.5	0.5	64	64	64	64	64	2	2	2	2	2
MRSA	NRS 119	Resistant		2	2	1	2	2	4	4	2	4	4	0.25	0.25	0.5	0.5	0.5	1	1	1	1	1	2	2	1	1	2
MRSA	NRS 384	Resistant		1	1	1	1	1	2	2	2	1	2	0.5	0.25	0.25	0.5	0.5	0.03	0.06	0.06	0.06	0.06	2	2	1	1	2
MRSA	581101692:1	Clinical Isolate; Resistant		1	2	2		2	2	4	4		4	0.5	0.5	0.5		0.5	64	64	64		64	2	2	2		2
MRSA	581101692:2	Clinical Isolate; Resistant		4	2	2	2	2	4	2	4	4	4	0.5	0.5	0.25	0.5	0.5	64	64	64	64	64	4	2	2	2	2
MRSA	581101692:3	Clinical Isolate; Resistant		2	1	2	2	2	2	4	4	4	4	0.5	0.5	0.25	0.5	0.5	64	64	64	64	64	2	2	2	2	2
MRSA	50316-0509	Clinical Isolate; Resistant		1	1	1	1	1	2	2	2	2	2	0.25	0.25	0.25	0.5	0.25	4	4	0.125	0.125	4	2	2	2	2	2
MRSA	51418-7407	Clinical Isolate; Resistant		1	1	1		1	1	2	1		1	0.5	0.5	0.5		0.5	0.125	0.125	0.125		0.125	2	2	2		2
MRSA	49496-1320	Clinical Isolate; Resistant		1	1	1		1	1	1	2	1	1	0.25	0.5	0.5	0.5	0.5	0.06	0.06	0.125	0.125	0.125	2	2	2	2	2
MRSA	M33376	Clinical Isolate; Resistant	Fluid Peritoneal	0.5	1	1	1	1	2	2	2	2	2	64	64	64	64	64	64	64	64	64	64	2	2	2	2	2
MRSA	M35249	Clinical Isolate; Resistant	ETA	1	1	1		1	1	2	2		2	0.25	0.25	0.25		0.25	0.03	0.06	0.125		0.125	2	1	1		1
MRSA	M38184	Clinical Isolate; Resistant	Wound	1	1	2	1	1	1	1	2	2	2	0.25	0.25	0.25	0.25	0.25	0.125	0.125	0.125	0.125	0.125	2	2	4	2	2
MRSA	M31414	Clinical Isolate; Resistant	Sternal Wd	1	1	1	1	1	2	2	1	2	2	0.06	0.125	0.25	0.5	0.125	64	64	64	64	64	1	2	2	2	2
MRSA	M38509	Clinical Isolate; Resistant	Abscess	1	0.5	0.5		0.5	1	1	1		1	0.25	0.25	0.25		0.25	0.03	0.03	0.06		0.03	2	2	2		2

MRSA	M39864	Clinical Isolate; Resistant	Wound	1	1	1	1	1	1	2	1	1	1	0.25	0.25	0.25	0.25	0.25	0.03	0.06	0.03	0.03	0.03	1	1	2	1	1
MRSA	M40725	Clinical Isolate; Resistant	Wound	1	1	1	1	1	2	2	1	1	2	2	1	1	1	1	0.03	0.06	0.03	0.03	0.03	2	2	2	2	2
MRSA	M45447	Clinical Isolate; Resistant	CSF	1	1	1	1	1	1	1	1	1	1	0.25	0.25	0.25	0.25	0.25	0.06	0.03	0.06	0.06	0.06	4	2	2	2	2
MRSA	M48439	Clinical Isolate; Resistant	Abscess	1	1			1	1	1			1	0.25	0.25			0.25	0.03	0.03			0.03	2	4			4
MRSA	M49406	Clinical Isolate; Resistant	Wound	1	1	1	1	1	1	2	1	1	1	0.25	0.25	0.5	0.5	0.5	0.125	0.125	64	0.06	0.125	4	2	2	2	2
MRSA	M51977	Clinical Isolate; Resistant	Wound	1	1	1	1	1	2	2	2	2	2	0.25	0.25	0.5	0.5	0.5	64	64	64	64	64	2	2	2	2	2
MRSA	M52817	Clinical Isolate; Resistant	Wound	1	1	1	1	1	2	2	1	2	2	0.25	0.25	0.25	0.25	0.25	0.03	0.125	0.125	0.06	0.125	2	4	2	2	2
MRSA	M54307	Clinical Isolate; Resistant	Wound	1	1	1		1	0.125	1	1		1	0.03	0.5	0.5		0.5	0.03	0.125	0.125		0.125	4	2	2		2
MRSA	M53519	Clinical Isolate; Resistant	Abscess	0.5	1	1	1	1	4	1	1	1	1	64	64	64	64	64	8	0.03	0.125	0.06	0.125	4	4	2	2	4
MRSA	M55707	Clinical Isolate; Resistant	Wound	1	1	1	1	1	2	2	1	2	2	0.25	0.25	0.5	0.25	0.25	0.125	0.03	0.125	0.06	0.125	4	2	2	2	2
MRSA	M56123	Clinical Isolate; Resistant	Wound	1	1	1	1	1	4	2	1	1	2	0.25	0.5	0.5	0.5	0.5	32	0.125	0.125	0.125	0.125	2	2	2	2	2
MRSA	M48662	Clinical Isolate; Resistant	Ulcer	1	1			1	1	1			1	0.5	0.5			0.5	64	64			64	2	2			2
MRSA	M49378	Clinical Isolate; Resistant	Wound	1	1			1	2	1			2	0.25	0.25			0.25	0.06	0.125			0.125	4	2			4
MRSA	M49411	Clinical Isolate; Resistant	Wound	1	1	1	1	1	1	2	1	1	1	0.125	0.25	0.25	0.25	0.25	0.03	0.06	0.03	0.03	0.03	2	4	2	2	2
MRSA	M56924	Clinical Isolate; Resistant	Wound	1	1			1	1	2			2	0.5	0.25			0.5	0.125	0.125			0.125	2	1			2
MRSA	M57543	Clinical Isolate; Resistant	Wound	1	2			2	2	2			2	0.25	0.5			0.5	64	64			64	4	4			4
MRSA	M57544	Clinical Isolate; Resistant	Wound	1	1	1	1	1	1	2	1	1	1	0.25	0.25	0.5	0.5	0.5	0.06	0.06	0.06	0.06	0.06	4	4	2	2	4
MRSA	M59014	Clinical Isolate; Resistant	Wound	1	1	1		1	2	2	1		2	0.25	0.25	0.25		0.25	0.125	0.06	0.125		0.125	4	4	2		4
MRSA	M60609	Clinical Isolate; Resistant	Wound	1	1	1	1	1	4	1	2	1	1	0.25	0.25	0.25	0.5	0.25	0.03	0.03	0.125	0.06	0.125	2	4	2	1	2
MRSA	M76385	Clinical Isolate; Resistant	Wound	1	1			1	2	1			2	4	0.5			4	8	4			8	4	2			4
MRSA	M61448	Clinical Isolate; Resistant	Wound	1	1	1		1	2	2	2		2	0.25	0.25	0.25		0.25	0.06	0.03	0.06		0.06	0.5	2	1		1
MRSA	M63450	Clinical Isolate; Resistant	Wound	1	2	1		1	2	2	0.25		2	0.5	0.25	0.5		0.5	64	64	64		64	2	2	2		2
MRSA	M74145	Clinical Isolate; Resistant	Tissue	1	1	1	1	1	4	4	4	4	4	0.25	0.25	0.25	0.5	0.25	1	1	1	1	1	8	8	8	8	8
MRSA	M74568	Clinical Isolate; Resistant	Ulcer	1	1	1	1	1	2	2	1	1	2	0.5	0.25	0.25	0.25	0.25	0.06	0.125	0.03	0.03	0.125	2	2	2	2	2
MRSA	M75365	Clinical Isolate; Resistant	Abscess	1	1			1	2	1			2	0.25	0.25			0.25	0.03	0.03			0.03	4	2			4
MRSA	M76558	Clinical Isolate; Resistant	Wd	2	1	1	2	2	2	2	2	2	2	0.25	0.5	0.5	0.5	0.5	64	64	64	64	64	4	2	2	2	2
MRSA	M77399	Clinical Isolate; Resistant	Tissue	1	1	1	1	1	2	4	2	2	2	64	64	64	64	64	0.06	0.06	0.125	0.25	0.125	4	2	2	2	2
MRSA	M78036	Clinical Isolate; Resistant	Wound	1	1	1		1	2	2	1		2	0.25	0.25	0.25		0.25	0.125	0.06	0.125		0.125	2	4	2		2
MRSA	M78540	Clinical Isolate; Resistant	Wound	1	1	1	1	1	2	2	2	2	2	0.125	0.25	0.5	0.5	0.5	0.03	0.06	0.125	0.125	0.125	4	4	2	2	4
MRSA	M81239	Clinical Isolate; Resistant	Wound	1	1	1	1	1	2	2	1	1	2	0.5	0.5	0.5	0.25	0.5	0.03	0.06	0.03	0.03	0.03	4	4	2	2	4
MRSA	M81986	Clinical Isolate; Resistant	Wound	1	1	1	1	1	1	2	1	1	1	0.5	0.5	0.5	0.25	0.5	0.06	0.03	0.125	0.03	0.125	4	4	2	2	4
MRSA	M82747	Clinical Isolate; Resistant	Wound	1	1	1	1	1	2	2	1	1	2	0.125	0.25	0.5	0.125	0.5	0.03	0.06	0.03	0.06	0.06	4	4	2	2	4
MRSA	M85049	Clinical Isolate; Resistant	Wound	1	1	1		1	2	1	1		1	0.5	0.06	0.25		0.25	0.03	0.03	0.125		0.03	4	2	2		2
MRSA	M85511	Clinical Isolate; Resistant	Wound	2	2	1	1	2	2	2	1	1	2	0.25	0.25	0.25	0.25	0.25	64	64	64	64	64	2	2	2	2	2
MRSA	M78411	Clinical Isolate; Resistant	Abscess	1	1			1	2	2			2	0.5	0.5			0.5	0.03	0.125			0.125	4	2			4
MRSA	M87512	Clinical Isolate; Resistant	Wound	1	1	1	1	1	2	2	1	1	2	0.5	0.5	0.5	0.5	0.5	0.06	0.06	0.06	0.03	0.06	4	4	2	2	4
MRSA	M90736	Clinical Isolate; Resistant	Wound	2	2			2	2	2			2	0.25	0.25			0.25	64	64			64	4	4			4
MRSA	M89569	Clinical Isolate; Resistant	Wound	1	1	1		1	1	2	1		1	0.5	0.5	0.5		0.5	64	64	64		64	2	2	2		2
MRSA	M88418	Clinical Isolate; Resistant	Wound	1	1	1		1	2	2	1		2	0.25	0.25	0.25		0.25	0.03	0.06	0.06		0.06	2	2	2		2
MRSA	M88210	Clinical Isolate; Resistant	Wound	1	1	1	1	1	1	2	1	1	1	0.5	0.5	0.5	0.5	0.5	0.06	0.06	8	0.06	0.06	2	2	1	1	2
MRSA	M97784	Clinical Isolate; Resistant	Abscess	1	1	1		1	2	1	1		1	0.25	0.5	0.5		0.5	0.125	0.06	0.06		0.06	4	2	2		2
MRSA	M97166	Clinical Isolate; Resistant	Wound	1	1			1	1	1			1	0.5	0.5			0.5	0.125	0.125			0.125	2	2			2
MRSA	M96912	Clinical Isolate; Resistant	Wound	1	1	1		1	2	0.5	0.5		0.5	0.5	0.5	0.5		0.5	64	64	64		64	4	2	2		2
MRSA	M234215	Clinical Isolate; Resistant	Sputum	1	1	1		1	2	0.5	0.5		0.5	0.5	0.5	0.5		0.5	0.125	0.125	0.125		0.125	4	2	2		2
MRSA	M121493	Clinical Isolate; Resistant	Wound	2	2	1	1	2	4	4	8	4	4	0.5	0.25	0.25	0.5	0.5	64	64	64	32	64	4	2	4	2	4
MRSA	M69739	Clinical Isolate; Resistant	Wound	1	1	1	1	1	2	2	0.5	0.5	2	0.5	0.5	0.5	0.25	0.5	0.06	0.06	0.06	0.06	0.06	4	4	2	2	4
MRSA	M69740	Clinical Isolate; Resistant	Abscess	1	1	1	1	1	2	2	2	4	2	0.5	0.125	0.25	0.25	0.25	64	64	64	64	64	4	4	2	2	4
MRSA	M70241	Clinical Isolate; Resistant	Wound	1	1	1		1	0.5	1	1		1	64	0.25	0.25		0.25	0.06	0.06	0.06		0.06	2	1	2		2
MRSA	M70964	Clinical Isolate; Resistant	Wound	1	1	1	1	1	1	2	1	1	1	0.25	0.25	0.5	0.25	0.25	0.06	2	0.06	0.06	0.06	2	2	1	1	2

MRSA	M71121	Clinical Isolate; Resistant	Abscess	1	1	1	1	1	1	0.5	1	1	1	1	0.5	0.25	0.25	1	2	0.125	0.03	0.6	0.125	2	2	2	2	2
MRSA	M71122	Clinical Isolate; Resistant	Wound	1	1	1	1	1	0.5	0.5	1	1	1	0.06	0.125	0.125	0.25	0.125	0.03	0.03	0.06	0.03	0.03	1	2	1	2	2
MRSA	M72749	Clinical Isolate; Resistant	Abscess	1	1	1	1	1	0.5	0.5	1	1	1	0.5	0.25	0.5	0.5	0.5	0.25	0.125	0.125	0.125	0.125	2	2	2	2	2
MRSA	M72760	Clinical Isolate; Resistant	Urine	1	1	2	1	1	8	8	4	4	8	0.125	1	0.125	0.125	0.125	64	64	64	64	64	2	4	2	2	2
MRSA	M73508	Clinical Isolate; Mutant	Abscess	1	1	1	1	1	1	1	1	1	1	0.125	0.25	0.5	0.125	0.5	0.125	0.125	0.125	0.06	0.125	2	2	1	1	2
MRSA	M74801	Clinical Isolate; Resistant	Wound	0.5	0.5	1	1	1	0.5	0.5	1	1	1	0.25	0.5	0.5	0.5	0.5	0.125	0.06	0.125	0.125	0.125	2	2	2	2	2
MRSA	M74804	Clinical Isolate; Resistant	Wound	1	1			1	1	1			1	0.5	0.5			0.5	0.06	0.125			0.125	2	2			2
MRSA	M64647	Clinical Isolate; Resistant	Wound	0.5	0.5	1	1	1	1	1	2	1	1	0.5	0.5	1	0.5	0.5	0.06	0.06	0.125	0.125	0.125	2	2	2	2	2
MRSA	M65412	Clinical Isolate; Resistant	Wound	1	1	1	1	1	2	2	1	1	2	0.5	0.25	0.25	0.25	0.25	0.125	0.125	0.125	0.125	0.125	2	2	1	1	2
MRSA	M65412	Clinical Isolate; Resistant	Abscess	0.5	1	1	1	1	0.5	0.5	1	1	1	0.5	0.5	0.5	0.5	0.5	0.125	0.125	0.06	0.06	0.125	2	2	2	2	2
MRSA	M66471	Clinical Isolate; Resistant	Abscess	1	1	1	1	1	1	1			1	0.5	0.25			0.5	0.06	0.06			0.06	1	1			1
MRSA	M66723	Clinical Isolate; Resistant	Abscess	1	1	1	1	1	1	0.5	1	1	1	0.25	0.25	0.25	0.25	0.25	0.06	0.125	0.06	0.06	0.06	2	2	2	1	2
MRSA	M67645	Clinical Isolate; Resistant	Abscess	1	1	1	1	1	1	2	1	1	1	0.5	0.5	0.25	0.25	0.5	0.125	0.125	0.06	0.125	0.125	2	2	1	2	2
MRSA	M67826	Clinical Isolate; Resistant	Abscess	1		1	1	1	0.5		1	1	1	0.25	0.5	0.25		0.25	0.03	0.06	0.03		0.03	2	2	2		2
MRSA	M67934	Clinical Isolate; Resistant	Abscess	1	1	1	1	1	2	0.5	1	1	1	0.25	0.25	0.25	0.25	0.25	0.25	0.06	0.125	0.125	0.125	2	2	1	2	2
MRSA	M68334	Clinical Isolate; Resistant	Abscess	1	1	1	1	1	1	0.5	2	2	2	0.125	0.125	0.5	0.5	0.5	0.06	0.03	0.06	0.06	0.06	2	2	2	1	2
MRSA	M69124	Clinical Isolate; Resistant	Sputum	1	0.5	1	1	1	1	0.5	1	1	1	0.25	0.25	0.25	0.25	0.25	0.03	0.125	0.06	0.06	0.06	2	2	1	1	2
MRSA	M72169	Clinical Isolate; Resistant	Wound	1	1	1	1	1	2	2	2	2	2	0.5	0.5	0.5	0.5	0.5	0.125	0.125	0.125	0.125	0.125	2	2	2	2	2
MRSA	M72746	Clinical Isolate; Resistant	Pus	1		1	1	1	1		2	1	1	0.5		0.5	0.5	0.5	0.125	0.125	0.125		0.125	2	2	2		2
MRSA	M73705	Clinical Isolate; Mutant	Wound	1	1	1	1	1	1	0.5	1	1	1	0.25	0.5	0.5	0.5	0.5	0.125	0.03	0.125	0.125	0.125	2	2	1	2	2
MRSA	M75392	Clinical Isolate; Resistant	Abscess	1	1	1	1	1	0.5	1	2	2	2	0.5	2	0.5	0.5	0.5	0.25	8	0.125	0.125	0.125	2	2	1	1	2
MRSA	M75683	Clinical Isolate; Resistant	Wound	1	1	1	1	1	1	0.5	2	2	2	0.25	0.25	0.5	0.5	0.5	0.06	0.06	0.125	0.06	0.06	2	2	1	1	2
MRSA	M75856	Clinical Isolate; Resistant	Wound	1	1	1	1	1	0.5	1	2	2	2	0.125	0.125	0.5	0.5	0.5	0.06	0.03	0.03	0.125	0.125	2	2	1	1	2
MRSA	M75899	Clinical Isolate; Resistant	Abscess	0.5	1	1	1	1	0.5	0.5	2	1	2	0.25	0.5	0.5	0.5	0.5	64	64	64	64	64	2	2	1	2	2
MRSA	M76067	Clinical Isolate; Resistant	Wound	1	1	1	1	1	2	2	2	2	2	0.125	0.125	0.5		0.125	64	64	64	64	64	2	2	2	2	2
MRSA	M76386	Clinical Isolate; Resistant	Abscess	1	1	1	1	1	0.5	0.5	2	2	2	0.25	0.25	0.25	0.25	0.25	0.06	0.06	64	64	64	2	2	1	2	2
MRSA	ATCC 43300	Induced Daptomycin Resist	-	1	0.5	1	1	1	16	16	16	16	16	0.25	0.25	0.5	0.5	0.5	64	64	64	64	64	0.5	0.25	0.25	0.25	0.25
MRSA	ATCC 43300	Induced Linezolid Resist	-	2	2	2	2	2	4	4	1	4	4	0.25	0.25	0.5	0.5	0.5	64	64	64	64	64	2	2	2	1	2
MRSA	ATCC 43300	Induced Dalbavancin Resist	-	2	2	1	1	2	4	4	1	2	4	0.25	0.25	0.25	0.5	0.25	64	64	64	64	64	2	2	1	1	2
MRSA	ATCC 6538;	FDA 209	-	1	1	1	1	1	0.5	0.5	1	1	1	0.125	0.125	0.25	0.25	0.25	0.06	0.06	0.03	0.03	0.06	2	2	1	1	2
MRSA	ATCC 43300	Induced CBD Resist	-	2	2	2	2	2	1	2	2	2	2	0.5	0.5	0.5	0.5	0.5	64	64	64	64	64	2	2	2	2	2
VISA	NRS 1; Mu50; ATCC 700699	Resistant (high level GISA)		8	8	8	8	8	8	8	8	8	8	0.5	0.25	0.25	0.25	0.25	64	64	64	64	64	2	2	2	2	2
VISA	NRS 2; ATCC 700698	Resistant		2	2	1	1	2	4	4	4	2	4	0.5	0.5	0.25	0.25	0.5	64	64	64	64	64	2	2	2	2	2
VISA	NRS 17	Resistant		8	8	8	8	8	8	8	8	8	8	0.5	0.5	0.5	0.5	0.5	0.125	0.125	0.125	0.125	0.125	2	2	2	2	2
VISA	NRS 18	Resistant		4	4	4	8	4	4	4	4	8	4	16	16	16	16	16	64	64	64	64	64	2	2	2	2	2
VISA	NRS 19	Resistant		4	2	2	4	4	4	4	4	4	4	1	1	1	1	1	64	64	64	64	64	2	2	1	1	2
VRSA	VRS3b	Resistant		8	16	4	4	8	1	1	1	1	1	0.25	0.25	0.25	0.25	0.25	64	64	64	64	64	2	2	1	1	2
VRSA	VRS4	Resistant		64	64	16	8	64	4	2	2	2	2	0.25	0.25	0.5	0.5	0.5	64	64	64	64	64	2	2	1	1	2
VRSA	VRS1	Resistant		64	64	64	64	64	2	2	2	2	2	32	32	16	16	32	64	64	64	64	64	2	2	1	1	2
VRSA	VRS10	Resistant		64	64	64	64	64	2	2	4	2	2	0.5	0.5	0.25	0.5	0.5	64	64	64	64	64	2	2	1	1	2

Supplementary Table 3. *S. aureus* and beta-hemolytic streptococci MIC90 summary
(Micromyx Isolates, µg mL^{-1}, n = 1)

Organism	Drug	MIC Range			Mode	MIC50	MIC90	%S	%R
Staphylococcus aureus (100)	CBD	1	-	2	1	1	1	NA	NA
	CLI	≤0.015	-	>16	0.12	0.12	>16	83.0%	17.0%
	ERY	0.25	-	>16	>16	>16	>16	42.0%	56.0%
	LVX	0.03	-	>16	0.12, 0.25	0.25	>16	55.0%	44.0%
	MUP	≤0.25	-	>256	≤0.25	≤0.25	≤0.25	NA	NA
	OXA	0.12	-	>32	>32	2	>32	50.0%	50.0%
	RET	≤0.03	-	0.12	≤0.03	≤0.03	0.12	NA	NA
	VAN	0.25	-	4	0.5	0.5	1	98.0%	0.0%
subset of MSSA (50)	CBD	1	-	2	1	1	1	NA	NA
	CLI	≤0.015	-	>16	0.12	0.12	0.25	92.0%	8.0%
	ERY	0.25	-	>16	0.25	0.5	>16	70.0%	28.0%
	LVX	0.03	-	>16	0.12	0.12	0.5	90.0%	10.0%
	MUP	≤0.25	-	≤0.25	≤0.25	≤0.25	≤0.25	NA	NA
	OXA	0.12	-	2	0.25	0.5	1	100%	0.0%
	RET	≤0.03	-	0.12	≤0.03	≤0.03	0.12	NA	NA
	VAN	0.25	-	1	0.5	0.5	1	100%	0.0%
subset of MRSA (50)	CBD	1	-	2	1	1	1	NA	NA
	CLI	0.06	-	>16	0.12	0.12	>16	74.0%	26.0%
	ERY	0.25	-	>16	>16	>16	>16	14.0%	84.0%
	LVX	0.12	-	>16	4	4	>16	20.0%	78.0%
	MUP	≤0.25	-	>256	≤0.25	≤0.25	32	NA	NA
	OXA	4	-	>32	>32	>32	>32	0.0%	100%
	RET	≤0.03	-	0.12	≤0.03	≤0.03	0.12	NA	NA
	VAN	0.25	-	4	0.5	0.5	1	96.0%	0.0%
Beta-hemolytic streptococci (59)[1]	CBD	8	-	32	16	16	16	NA	NA
	CLI	0.03	-	>16	0.03	0.06	>16	72.9%	27.1%
	ERY	≤0.015	-	>16	0.03, 0.06	0.06	>16	57.6%	42.4%
	LVX	0.12	-	2	0.5	0.5	1	100%	0.0%
	MUP	≤0.25	-	2	≤0.25	≤0.25	1	NA	NA
	OXA	≤0.03	-	0.5	≤0.03	0.25	0.5	NA	NA
	RET	≤0.03	-	0.06	≤0.03	≤0.03	≤0.03	NA	NA
	VAN	0.25	-	1	0.25	0.25	0.5	100%	0.0%
subset of *Streptococcus pyogenes* (29)	CBD	8	-	32	16	16	16	NA	NA
	CLI	0.03	-	>16	0.03	0.06	>16	89.7%	10.3%
	ERY	≤0.015	-	>16	0.03	0.06	>16	86.2%	13.8%
	LVX	0.12	-	2	0.5	0.5	1	100%	0.0%
	MUP	≤0.25	-	0.5	≤0.25	≤0.25	≤0.25	NA	NA
	OXA	≤0.03	-	0.12	≤0.03	≤0.03	0.06	NA	NA
	RET	≤0.03	-	0.06	≤0.03	≤0.03	≤0.03	NA	NA
	VAN	0.25	-	1	0.25	0.25	0.5	100%	0.0%
subset of *Streptococcus agalactiae* (30)	CBD	8	-	32	16	16	16	NA	NA
	CLI	0.03	-	>16	>16	0.06	>16	56.7%	43.3%
	ERY	0.03	-	>16	>16	2	>16	30.0%	70.0%
	LVX	0.5	-	1	0.5	0.5	1	100%	0.0%
	MUP	≤0.25	-	2	≤0.25	≤0.25	1	NA	NA
	OXA	0.25	-	0.5	0.25	0.25	0.5	NA	NA
	RET	≤0.03	-	≤0.03	≤0.03	≤0.03	≤0.03	NA	NA
	VAN	0.25	-	0.5	0.5	0.5	0.5	100%	0.0%

CLI, clindamycin; ERY, erythromycin, LVX, levofloxacin; MUP, mupirocin; OXA, oxacillin; RET, retapamulin; VAN, vancomycin; MSSA, methicillin-susceptible *S. aureus*; MRSA, methicillin-resistant *S. aureus*; NA, not applicable; %S, percentage of isolates that are susceptible according to established CLSI breakpoints; %R, percentage of isolates that are resistant according to established CLSI breakpoints; parentheses denote number of isolates tested in each group

Supplementary Table 4. ***S. aureus*** **MIC90** (Micromyx Isolate individual MIC)

**MMX number**	**Pheno-type**	**MIC (µg mL^{-1})** (n = 1)												
		**CBD**	**CLI**		**ERY**		**LVX**		**MUP**	**OXA**		**RET**	**VAN**	
7793	MSSA	1	0.12	S	0.5	S	0.25	S	≤0.25	0.25	S	0.06	1	S
7794	MSSA	1	0.12	S	0.25	S	0.5	S	≤0.25	1	S	0.06	1	S
7795	MSSA	1	0.12	S	0.5	S	0.12	S	≤0.25	1	S	0.06	1	S
7797	MSSA	1	0.12	S	>16	R	0.5	S	≤0.25	1	S	0.12	1	S
7798	MSSA	1	0.12	S	0.25	S	0.12	S	≤0.25	0.5	S	0.06	0.5	S
7799	MSSA	1	0.12	S	0.25	S	0.12	S	≤0.25	1	S	0.06	0.25	S
7800	MSSA	1	0.12	S	0.25	S	0.03	S	≤0.25	0.25	S	0.06	0.5	S
7801	MSSA	1	0.12	S	>16	R	0.12	S	≤0.25	0.5	S	0.12	0.5	S
7802	MSSA	1	0.12	S	>16	R	16	R	≤0.25	0.5	S	0.06	0.5	S
7803	MSSA	1	0.12	S	0.25	S	0.25	S	≤0.25	1	S	0.12	1	S
7804	MSSA	1	0.12	S	0.25	S	0.25	S	≤0.25	1	S	0.06	1	S
7805	MSSA	1	0.12	S	0.25	S	0.25	S	≤0.25	0.25	S	0.12	0.5	S
7806	MSSA	1	0.12	S	0.25	S	0.12	S	≤0.25	0.5	S	0.06	0.5	S
7894	MSSA	1	>16	R	>16	R	4	R	≤0.25	0.5	S	≤0.03	1	S
7909	MSSA	1	0.06	S	0.25	S	0.12	S	≤0.25	0.25	S	≤0.03	1	S
7910	MSSA	1	0.06	S	0.25	S	0.25	S	≤0.25	0.25	S	≤0.03	1	S
7911	MSSA	1	0.06	S	0.25	S	0.12	S	≤0.25	0.12	S	≤0.03	0.5	S
7912	MSSA	1	0.25	S	0.5	S	0.25	S	≤0.25	0.25	S	≤0.03	0.5	S
7913	MSSA	1	0.06	S	0.25	S	0.25	S	≤0.25	0.5	S	≤0.03	1	S
7914	MSSA	2	0.12	S	16	R	8	R	≤0.25	1	S	≤0.03	1	S
7915	MSSA	1	0.06	S	0.5	S	0.25	S	≤0.25	0.25	S	≤0.03	0.5	S
7916	MSSA	1	0.06	S	0.25	S	0.12	S	≤0.25	0.25	S	≤0.03	0.5	S
7917	MSSA	1	0.06	S	0.25	S	0.12	S	≤0.25	0.5	S	≤0.03	0.5	S
7918	MSSA	1	0.12	S	0.5	S	0.25	S	≤0.25	0.5	S	≤0.03	1	S
7919	MSSA	1	>16	R	>16	R	>16	R	≤0.25	0.25	S	≤0.03	1	S
7920	MSSA	1	0.06	S	0.5	S	0.06	S	≤0.25	0.25	S	≤0.03	0.5	S
7921	MSSA	1	0.06	S	0.25	S	0.12	S	≤0.25	0.25	S	≤0.03	0.5	S
7922	MSSA	1	0.06	S	0.5	S	0.25	S	≤0.25	0.25	S	≤0.03	0.5	S
7923	MSSA	1	0.12	S	0.5	S	0.25	S	≤0.25	0.12	S	≤0.03	1	S
8834	MSSA	1	0.06	S	>16	R	16	R	≤0.25	1	S	≤0.03	0.5	S
8836	MSSA	1	0.12	S	>16	R	0.12	S	≤0.25	0.5	S	≤0.03	0.5	S
8838	MSSA	1	0.12	S	0.5	S	0.12	S	≤0.25	0.5	S	≤0.03	0.5	S
8839	MSSA	1	0.12	S	0.5	S	0.12	S	≤0.25	0.25	S	≤0.03	0.5	S
8840	MSSA	1	0.06	S	0.25	S	0.12	S	≤0.25	0.5	S	≤0.03	0.5	S
9093	MSSA	1	>16	R	>16	R	0.06	S	≤0.25	1	S	≤0.03	0.5	S
9094	MSSA	1	0.06	S	0.25	S	0.12	S	≤0.25	0.5	S	≤0.03	0.5	S
9095	MSSA	1	0.12	S	>16	R	0.12	S	≤0.25	0.5	S	≤0.03	0.5	S
9116	MSSA	1	≤0.015	S	0.25	S	0.25	S	≤0.25	0.12	S	≤0.03	0.5	S
9179	MSSA	1	0.06	S	0.5	S	0.12	S	≤0.25	0.25	S	≤0.03	0.5	S
9180	MSSA	1	0.12	S	8	R	0.12	S	≤0.25	1	S	≤0.03	0.5	S
9181	MSSA	1	0.06	S	0.25	S	0.12	S	≤0.25	0.5	S	≤0.03	1	S
9182	MSSA	1	0.12	S	8	R	0.12	S	≤0.25	1	S	≤0.03	0.5	S
9183	MSSA	1	0.25	S	0.5	S	0.12	S	≤0.25	0.5	S	≤0.03	0.5	S
9184	MSSA	1	0.12	S	0.5	S	0.25	S	≤0.25	0.25	S	≤0.03	1	S
9185	MSSA	1	0.12	S	0.5	S	0.25	S	≤0.25	0.25	S	≤0.03	0.5	S
9233	MSSA	1	0.12	S	0.25	S	0.12	S	≤0.25	0.25	S	≤0.03	0.5	S
10714	MSSA	1	0.25	S	1	I	0.25	S	≤0.25	2	S	0.12	1	S
10716	MSSA	1	0.25	S	0.5	S	0.25	S	≤0.25	1	S	0.12	1	S
10718	MSSA	1	0.25	S	>16	R	0.25	S	≤0.25	1	S	0.12	1	S
10719	MSSA	1	>16	R	>16	R	0.25	S	≤0.25	0.5	S	0.06	1	S

MMX number	Pheno-type	MIC (μg mL^{-1})													
		CBD	CLI		ERY		LVX		MUP	OXA		RET	VAN		
7774	MRSA	1	0.12	S	>16	R	>16	R	≤0.25	>32	R	0.06	0.5	S
7775	MRSA	1	0.12	S	>16	R	16	R	≤0.25	>32	R	0.12	0.5	S
7776	MRSA	1	>16	R	>16	R	4	R	≤0.25	>32	R	≤0.03	0.5	S
7777	MRSA	1	>16	R	>16	R	>16	R	≤0.25	>32	R	0.06	1	S
7778	MRSA	1	0.25	S	>16	R	0.12	S	16	>32	R	0.06	0.5	S
7779	MRSA	1	0.12	S	>16	R	4	R	>256	>32	R	0.06	0.5	S
7780	MRSA	2	0.12	S	0.5	S	16	R	≤0.25	>32	R	0.06	1	S
7781	MRSA	1	0.12	S	0.25	S	4	R	≤0.25	>32	R	0.06	0.5	S
7782	MRSA	1	0.12	S	>16	R	4	R	>256	>32	R	0.06	0.5	S
7783	MRSA	1	0.12	S	16	R	4	R	≤0.25	16	R	0.12	0.5	S
7784	MRSA	1	>16	R	>16	R	>16	R	≤0.25	>32	R	0.12	0.5	S
7785	MRSA	1	0.12	S	>16	R	8	R	≤0.25	>32	R	0.06	0.5	S
7786	MRSA	1	>16	R	>16	R	8	R	≤0.25	>32	R	0.12	0.5	S
7787	MRSA	1	0.12	S	>16	R	4	R	≤0.25	>32	R	0.06	0.5	S
7788	MRSA	1	0.25	S	>16	R	16	R	≤0.25	>32	R	0.06	1	S
7792	MRSA	1	0.12	S	1	I	4	R	≤0.25	4	R	0.06	1	S
7796	MRSA	1	0.12	S	0.5	S	0.25	S	≤0.25	4	R	0.12	4	I
7892	MRSA	1	>16	R	>16	R	>16	R	4	>32	R	≤0.03	0.5	S
7893	MRSA	1	0.06	S	>16	R	8	R	≤0.25	>32	R	≤0.03	0.5	S
7895	MRSA	1	0.12	S	>16	R	4	R	≤0.25	>32	R	≤0.03	0.5	S
7896	MRSA	1	0.12	S	>16	R	4	R	≤0.25	>32	R	≤0.03	0.5	S
7897	MRSA	1	>16	R	>16	R	>16	R	≤0.25	>32	R	≤0.03	1	S
7898	MRSA	2	0.12	S	>16	R	0.25	S	≤0.25	>32	R	≤0.03	1	S
7899	MRSA	1	0.06	S	>16	R	0.12	S	≤0.25	>32	R	≤0.03	0.25	S
7900	MRSA	1	0.06	S	0.25	S	0.12	S	≤0.25	>32	R	≤0.03	0.5	S
7901	MRSA	1	0.06	S	>16	R	0.25	S	≤0.25	>32	R	≤0.03	0.5	S
7902	MRSA	1	>16	R	>16	R	>16	R	≤0.25	>32	R	≤0.03	1	S
7903	MRSA	1	>16	R	>16	R	16	R	≤0.25	>32	R	≤0.03	1	S
7904	MRSA	1	0.12	S	>16	R	4	R	≤0.25	>32	R	≤0.03	0.5	S
7905	MRSA	1	0.06	S	>16	R	0.25	S	≤0.25	>32	R	≤0.03	1	S
7906	MRSA	1	>16	R	>16	R	>16	R	32	>32	R	0.06	2	S
8841	MRSA	1	0.06	S	>16	R	>16	R	≤0.25	>32	R	≤0.03	0.5	S
8842	MRSA	1	>16	R	>16	R	16	R	≤0.25	32	R	≤0.03	0.5	S
8844	MRSA	1	0.06	S	0.25	S	0.25	S	≤0.25	>32	R	≤0.03	0.5	S
8845	MRSA	1	>16	R	>16	R	>16	R	>256	>32	R	≤0.03	1	S
9096	MRSA	1	0.06	S	>16	R	2	I	≤0.25	>32	R	≤0.03	1	S
9117	MRSA	1	>16	R	>16	R	>16	R	≤0.25	>32	R	≤0.03	1	S
9201	MRSA	1	0.06	S	>16	R	4	R	≤0.25	>32	R	≤0.03	1	S
9202	MRSA	1	>16	R	>16	R	>16	R	>256	>32	R	0.12	1	S
9203	MRSA	1	0.06	S	>16	R	4	R	≤0.25	>32	R	≤0.03	0.5	S
9204	MRSA	1	0.12	S	>16	R	4	R	≤0.25	>32	R	≤0.03	0.5	S
9210	MRSA	1	0.12	S	>16	R	4	R	≤0.25	32	R	≤0.03	0.5	S
9213	MRSA	1	0.06	S	>16	R	16	R	≤0.25	32	R	≤0.03	0.5	S
9216	MRSA	1	0.06	S	>16	R	0.25	S	≤0.25	32	R	≤0.03	0.5	S
10715	MRSA	1	0.12	S	>16	R	8	R	≤0.25	>32	R	0.06	1	S
10717	MRSA	1	0.12	S	>16	R	8	R	≤0.25	>32	R	0.12	1	S
10720	MRSA	1	0.12	S	>16	R	16	R	≤0.25	>32	R	0.06	4	I
10721	MRSA	1	0.12	S	0.5	S	8	R	>256	>32	R	≤0.03	1	S
10722	MRSA	1	0.12	S	>16	R	16	R	≤0.25	>32	R	0.06	1	S
10723	MRSA	1	0.12	S	0.5	S	0.25	S	≤0.25	16	R	0.06	1	S
10723	MRSA	1	0.12	S	0.5	S	0.25	S	≤0.25	16	R	0.06	1	S

MMX, Micromyx repository number; MRSA, methicillin-resistant *S. aureus;* S, susceptible by CLSI breakpoints; R, resistant by CLSI breakpoints; CLI, clindamycin; ERY, erythromycin; LVX, levofloxacin; MUP, mupirocin; OXA, oxacillin; RET, retapamulin; VAN, vancomycin

Supplementary Table 5. -hemolytic streptococci MIC90 (Micromyx isolate MIC)

MMX number	*Streptococcus pyogenes* MIC (μg mL^{-1}) (n = 1)											
	CBD	CLI		ERY		LVX		MUP	OXA	RET	VAN	
8781	8	0.06	S	0.03	S	0.5	S	≤0.25	≤0.03	≤0.03	0.25	S
8790	16	0.03	S	0.03	S	0.5	S	≤0.25	≤0.03	≤0.03	0.25	S
8817	16	0.03	S	0.03	S	0.25	S	≤0.25	≤0.03	≤0.03	0.25	S
8818	8	0.03	S	≤0.015	S	0.25	S	≤0.25	≤0.03	≤0.03	0.25	S
8819	8	0.03	S	0.03	S	0.25	S	≤0.25	≤0.03	≤0.03	0.25	S
8820	16	0.03	S	0.06	S	0.5	S	≤0.25	≤0.03	≤0.03	0.25	S
8821	8	0.03	S	0.03	S	0.5	S	≤0.25	≤0.03	≤0.03	0.25	S
8822	16	0.03	S	0.06	S	0.5	S	≤0.25	0.06	≤0.03	0.5	S
8823	8	0.06	S	>16	R	0.25	S	≤0.25	0.06	≤0.03	0.25	S
8824	16	0.03	S	0.03	S	0.25	S	≤0.25	0.06	≤0.03	0.25	S
8825	16	0.03	S	0.03	S	1	S	≤0.25	≤0.03	≤0.03	0.25	S
8827	16	0.03	S	0.03	S	0.5	S	≤0.25	≤0.03	≤0.03	0.25	S
8828	16	>16	R	>16	R	0.5	S	≤0.25	≤0.03	≤0.03	0.25	S
8829	8	>16	R	>16	R	0.5	S	0.5	≤0.03	≤0.03	0.25	S
8902	8	0.06	S	0.06	S	0.5	S	≤0.25	≤0.03	≤0.03	0.25	S
9170	16	0.06	S	0.03	S	0.5	S	≤0.25	≤0.03	≤0.03	0.25	S
9171	16	0.06	S	0.06	S	0.5	S	≤0.25	≤0.03	≤0.03	0.25	S
9172	16	0.03	S	0.03	S	1	S	≤0.25	≤0.03	≤0.03	0.25	S
9173	16	0.06	S	0.06	S	0.25	S	≤0.25	≤0.03	≤0.03	0.25	S
9174	8	0.06	S	0.06	S	0.5	S	≤0.25	≤0.03	≤0.03	0.25	S
9175	16	0.06	S	0.06	S	0.5	S	≤0.25	≤0.03	≤0.03	0.25	S
9176	32	0.12	S	0.12	S	0.12	S	≤0.25	0.12	0.06	1	S
9177	16	0.06	S	0.06	S	2	S	≤0.25	≤0.03	≤0.03	0.25	S
9178	16	0.06	S	0.06	S	2	S	≤0.25	0.06	≤0.03	0.25	S
10604	16	0.06	S	0.06	S	0.5	S	≤0.25	≤0.03	≤0.03	0.25	S
10605	16	0.06	S	0.06	S	1	S	≤0.25	0.06	≤0.03	0.5	S
10606	16	0.03	S	0.03	S	0.5	S	≤0.25	≤0.03	≤0.03	0.25	S
10607	16	>16	R	>16	R	0.5	S	≤0.25	≤0.03	≤0.03	0.25	S
10608	8	0.03	S	0.03	S	0.5	S	≤0.25	≤0.03	≤0.03	0.25	S

MMX number	*Streptococcus agalactiae* MIC (μg mL^{-1})											
	CBD	CLI		ERY		LVX		MUP	OXA	RET	VAN	
8696	16	0.06	S	0.06	S	0.5	S	≤0.25	0.25	≤0.03	0.5	S
8697	16	0.06	S	4	R	1	S	≤0.25	0.5	≤0.03	0.5	S
8698	16	0.03	S	0.03	S	0.5	S	≤0.25	0.25	≤0.03	0.25	S
8699	16	0.03	S	2	R	0.5	S	≤0.25	0.5	≤0.03	0.25	S
8700	16	0.03	S	0.03	S	0.5	S	≤0.25	0.25	≤0.03	0.25	S
8701	16	0.03	S	2	R	0.5	S	≤0.25	0.5	≤0.03	0.25	S
8702	16	0.06	S	2	R	0.5	S	≤0.25	0.5	≤0.03	0.5	S
8703	16	0.03	S	0.06	S	0.5	S	≤0.25	0.5	≤0.03	0.5	S
8704	16	0.03	S	0.03	S	0.5	S	≤0.25	0.25	≤0.03	0.5	S
8705	16	0.03	S	4	R	0.5	S	≤0.25	0.25	≤0.03	0.5	S
8786	16	>16	R	>16	R	0.5	S	1	0.5	≤0.03	0.25	S
8787	16	0.03	S	1	R	0.5	S	1	0.25	≤0.03	0.25	S
8788	8	>16	R	>16	R	1	S	0.5	0.5	≤0.03	0.25	S
8789	16	>16	R	>16	R	0.5	S	0.5	0.25	≤0.03	0.5	S
8791	16	>16	R	>16	R	0.5	S	1	0.25	≤0.03	0.5	S
8792	16	>16	R	1	R	1	S	2	0.5	≤0.03	0.5	S
8793	16	0.12	S	1	R	0.5	S	1	0.25	≤0.03	0.25	S
8794	16	0.03	S	0.03	S	0.5	S	1	0.25	≤0.03	0.5	S
8795	16	>16	R	>16	R	0.5	S	0.5	0.25	≤0.03	0.5	S
8796	16	0.06	S	0.06	S	1	S	1	0.25	≤0.03	0.5	S
8892	16	>16	R	>16	R	0.5	S	≤0.25	0.25	≤0.03	0.25	S
10609	16	>16	R	>16	R	0.5	S	≤0.25	0.25	≤0.03	0.25	S
10610	32	>16	R	8	R	1	S	1	0.5	≤0.03	0.5	S
10611	16	0.06	S	0.06	S	0.5	S	≤0.25	0.25	≤0.03	0.5	S
10612	16	>16	R	>16	R	0.5	S	≤0.25	0.25	≤0.03	0.5	S
10613	16	0.06	S	0.06	S	0.5	S	≤0.25	0.25	≤0.03	0.5	S
10729	16	0.03	S	2	R	0.5	S	≤0.25	0.25	≤0.03	0.25	S
10730	16	>16	R	>16	R	0.5	S	≤0.25	0.25	≤0.03	0.5	S
10731	16	>16	R	>16	R	0.5	S	≤0.25	0.25	≤0.03	0.5	S
10732	16	>16	R	>16	R	0.5	S	≤0.25	0.25	≤0.03	0.5	S

MMX, Micromyx repository number; S, susceptible by CLSI breakpoints; R, resistant by CLSI breakpoints; CLI, clindamycin; ERY, erythromycin; LVX, levofloxacin; MUP, mupirocin; OXA, oxacillin; RET, retapamulin; VAN, vancomycin

Supplementary Table 6: Minimum inhibitory concentrations (MICs) of CBD (μg mL^{-1}) against other classes of microorganisms (n = 4).

**Yeasts**	**Strain**	**Flucon-azole**	**5-fluoro-cytposine**			**CBD**
Candida albicans[1]	ATCC 90028	1	0.5-1			>128
Cryptococcus neoformans var. grubii[1] H99	ATCC 208821	8–16	0.5-1			128
**Mycobacteria**	**Strain**	**Mupirocin**	**Isoniazid**	**Linezolid**	**Rifam-picin**	**CBD**
Mycobacterium smegmatis Mc(2)155[1]	ATCC 700084	256	8	0.5	32	16
Mycobacterium tuberculosis H37Rv[1]			1		2	>64 (70% inhib)

[1]MIC assays at UQ

Supplementary Table 7. Minimum inhibitory concentrations (MICs) of CBD ($\mu g\ mL^{-1}$) against Gram-negative bacteria.

Species	Strain	Colistin	Trimeth-oprim	Mupir-ocin	Clinda-mycin	CBD
Escherichia coli[1]	ATCC 25922	0.06–0.125	0.5–2	>64	>64	>64
Klebsiella pneumoniae[1]	ATCC 700603	0.125–1	8–16	>64	>64	>64
Pseudomonas aeruginosa[1]	ATCC 27853	0.25–1	>64	>64	>64	>64
Neisseria gonorrhoeae 24h/48h[1]	ATCC 19424	32/32		≤0.03/0.03	1/1	2/2
	**Strain**	**Vanco-mycin**	**Levo-floxacin**	**Mero-penem**	**Genta-micin**	**CBD**
Campylobacter jejuni[2]	MMX 5491	>32	0.25	≤0.03	1	>64
Escherichia coli[2]	ATCC 25922	>32	0.015	≤0.03	0.25	>64
Escherichia coli[2]	MMX 9022 ESBL	>32	16	0.06	0.5	>64
Enterobacter cloacae[2]	MMX 6089	>32	0.015	≤0.03	0.12	>64
Enterobacter cloacae[2]	MMX 6308 MDR	>32	32	16	>32	>64
Klebsiella pneumoniae[2]	ATCC 13883	>32	0.12	≤0.03	0.25	>64
Klebsiella pneumoniae[2]	MMX 4623 KPC-2	>32	1	>32	>32	>64
Serratia marcescens[2]	MMX 6462	>32	0.06	0.12	1	>64
Serratia marcescens[2]	MMX 4544 MDR	>32	0.06	0.06	>32	>64
Stenotrophomonas maltophila[2]	MMX 4746	>32	0.5	8	0.5	>64
Stenotrophomonas maltophila[2]	MMX 4708 MDR	>32	1	>32	>32	>64
Burkholderia cepacia[2]	MMX 547 GM^R	>32	2	2	>32	>64
Proteus mirabilis[2]	MMX 6442	>32	0.015	≤0.03	0.25	>64
Proteus mirabilis[2]	MMX 4524 MDR	>32	8	0.06	8	>64
Salmonella typhimurium[2]	ATCC 35987	>32	0.03	≤0.03	0.25	>64
Salmonella Group C[2]	MMX 5238	>32	0.03	≤0.03	0.25	>64
Shigella dysenteriaev[2]	ATCC 29026	>32	0.03	≤0.03	0.5	>64
Shigella sonnei[2]	ATCC 25931	>32	0.015	≤0.03	0.5	>64
Morganella morganii[2]	MMX 6234	>32	0.03	0.06	0.5	>64
Morganella morganii[2]	MMX 6437 MDR	>32	32	0.06	4	>64
Providencia stuartii[2]	MMX 6630	>32	0.06	≤0.03	0.25	>64
Providencia stuartii[2]	MMX 6488 MDR	>32	4	≤0.03	1	>64
Pseudomonas aeruginosa[2]	ATCC 27853	>32	1	0.5	1	>64
Haemophilus influenzae[2]	ATCC 49247	>32	0.03	1	2	>64
Haemophilus influenzae[2]	MMX 7988 BL-+ve	>32	0.015	≤0.03	2	>64
Moraxella catarrhalis[2]	MMX 3782 BL+ve	>32	0.03	≤0.03	0.12	1
Acinetobacter baumannii[2]	ATCC 19606	>32	0.25	2	16	>64
Acinetobacter baumannii[2]	MMX 6334 MDR	>32	8	>32	>32	>64
Citrobacter freundii[2]	MMX 6077	>32	0.03	≤0.03	0.5	>64
Citrobacter freundii[2]	MMX 6602 MDR	>32	0.06	0.06	0.5	>64
Neisseria gonorrhoeae[2]	ATCC 49226	8	0.004	≤0.03	8	2
Neisseria gonorrhoeae[2]	MMX 6746 CIP^R	32	4	≤0.03	16	2
Neisseria gonorrhoeae[2]	MMX 6757 CTXNS	32	0.015	≤0.03	8	2
Neisseria meningitidis[2]	ATCC 13090	>32	0.008	≤0.03	32	1
Legionella pneumophila[2]	MMX 7515	0.12	0.25	0.5	2	1
**Gram-Negative: Anaerobic Conditions**						
**Species**	**Strain**			**Metro-nidazole**	**Clinda-mycin**	**CBD**
Bacteroides fragilis[2]	ATCC 25285			1	0.5	16
Bacteroides ovatus[2]	MMX 3506			4	2	>64
Prevotella bivia[2]	MMX 3447			4	>32	8
Veillonella parvula[2]	MMX 1272			2	0.06	>64
Fusobacterium nucleatum[2]	MMX 9914			0.06	≤0.03	>64

[1]MIC assays at UQ (n = 4), [2]MIC assays at Micromyx (n = 1)

ESBL, extended-spectrum beta-lactamase producing; KPC, Klebsiella pneumoniae carbapenemase; MDR, multi-drug resistant; BL-+ve, beta-lactamase positive; CIP^R, ciprofloxacin resistant; GM^R, gentamicin resistant; CTXNS, ceftriaxone non-susceptible

Supplementary Table 8. Minimum inhibitory concentrations (MICs) of CBD (μg mL^{-1}) and comparators against 30 isolates of *Neisseria gonorrhoeae* by broth microdilution assay (n = 1), and 26 isolates by agar dilution assay (n = 3).

Strain	Type	MIC (μg mL^{-1})						
		Broth Microdilution				Agar Dilution		
		CBD	Azithromycin	Ceftriaxone	Ciprofloxacin	CBD	Ceftriaxone	Ciprofloxacin
Staphylococcus aureus								
ATCC 29213	QC	0.5	0.5 (0.5-2)[1]	2 (1-8)[1]	0.25 (0.12-0.5)[1]			
Neisseria gonorrhoeae								
ATCC 49226	QC	2	0.25 (0.25-1)[1]	≤0.008 (0.016-0.004)[1]	≤0.008 (0.008-0.001)[1]	4	0.015 (0.004-0.015)	0.004 (0.001-0.008)
NCTC 13477	WHO F; CIP-S: AZI MIC 0.12	0.5	0.06	≤0.008	≤0.008	1	≤0.005	0.004
NCTC 13478	WHO G; CIP-I: AZI MIC 0.25	2	0.25	≤0.008	0.06	2	0.015	0.12
NCTC 13479	WHO K; CIP-R: AZI MIC 0.25	2	0.25	0.03	>8	1	0.06	>8
NCTC 13480	WHO L; CIP-R: AZI MIC 0.5	0.5	0.25	0.06	>8	2	0.25	>8
NCTC 13481	WHO M; CIP-R: AZI MIC 0.25	1	0.12	≤0.008	1	2	0.015	2
NCTC 13482	WHO N; CIP-R: AZI MIC 0.25	2	0.12	≤0.008	4	1	0.008	4
NCTC 13483	WHO O; CIP-S: AZI MIC 0.25	2	0.25	≤0.008	≤0.008	2	0.015	0.015
NCTC 13484	WHO P; CIP-S: AZI MIC 4	>32[2]	4	≤0.008	≤0.008	>32[2]	0.008	0.008
NCTC 13817	WHO U; CIP-S: AZI MIC 4	1	2	≤0.008	≤0.008	4	0.002	0.004
NCTC 13818	WHO V; CIP-R: AZI MIC >256	1	>8	≤0.008	>8	1	0.03	>8
NCTC 13819	WHO W; CIP-R, AZI MIC 0.5	1	0.25	0.015	>8	2	0.12	>8
NCTC 13820	WHO X; **CRO-NS**, CIP-R: AZI MIC 0.5	2	0.25	1	>8	2	2	>8
NCTC 13821	WHO Y; CIP-R: AZI MIC 1	1	0.5	0.5	>8	4	1	>8
MMX 10409	CIP-R	2	0.25	0.015	>8	2	0.03	>8
MMX 10410	CIP-S	2	0.12	≤0.008	≤0.008			
MMX 10447	CIP-S	1	0.06	≤0.008	≤0.008			
MMX 10450	CIP-S	0.5	0.12	≤0.008	≤0.008			
MMX 10452	CIP-R	2	0.12	≤0.008	>8			
MMX 10454	CIP-R	1	0.12	0.015	8			
MMX 6921	CIP-R	1	8	≤0.008	8			
MMX 6938	CIP-S	2	0.25	≤0.008	≤0.008			
CDC 0165	CIP-R: AZI MIC 1	8	1	0.015	>8	2	0.06	>8
CDC 0166	CIP-R: AZI MIC 1	2	0.5	0.015	8	2	0.06	>8
CDC 0167	CIP-S: AZI MIC 8	1	>8	≤0.008	≤0.008	2	0.008	0.004
CDC 0172	CIP-R: AZI MIC 0.5	2	0.25	≤0.008	8	2	0.06	>8
CDC 0174	NA					2	0.06	>8
CDC 0175	CIP-S: AZI MIC16	2	>8	≤0.008	≤0.008	2	0.008	0.004
CDC 0177	CIP-S: AZI MIC 2	2	2	≤0.008	≤0.008	2	0.015	0.015
CDC 0178	NA					2	0.06	>8
CDC 0180	NA					2	0.03	>8
CDC 0181	CIP-S: AZI MIC 256	1	>8	≤0.008	≤0.008	1	0.015	0.015
CDC 0194	CRO-NS, CIP-S: AZI MIC 0.5	2	0.25	0.03	≤0.008	2	0.25	0.004

[1]CLSI QC ranges shown in parentheses; [2]growth above 32 μg mL^{-1} was unable to be determined due to CBD precipitation.

AZI, azithromycin; CRO, ceftriaxone; CIP, ciprofloxacin; S, susceptible; R, resistant; NS, non-susceptible; NG, no growth

Supplementary Table 9. Minimum inhibitory concentrations (MICs) of antibiotics and CBD ($\mu g\ mL^{-1}$) against Gram-negative mutant bacteria.

<table>
<tr><th>Species</th><th colspan="4">Strain</th><th>CBD</th></tr>
<tr><td>Escherichia coli[1]</td><td colspan="4">Control strain, parent of mutant E. coli below</td><td>>128</td></tr>
<tr><td>Escherichia coli[1]</td><td colspan="4">DC2</td><td>>128</td></tr>
<tr><td>Escherichia coli[1]</td><td colspan="4">Deficient in lpxC</td><td>128</td></tr>
<tr><td>Escherichia coli[1]</td><td colspan="4">tolC efflux pump</td><td>>128</td></tr>
<tr><td>Escherichia coli[1]</td><td colspan="4">Mutant of lpxC and tolC</td><td>128</td></tr>
<tr><td>Pseudomonas aeruginosa[1]</td><td colspan="4">PAO1 expressing MexAB-OprM,</td><td>>128</td></tr>
<tr><td>Pseudomonas aeruginosa[1]</td><td colspan="4">Efflux pump mutant: d(mex-oprM) d(mexCD-oprJ) d(mexEF-oprN d(mexJKL) d(mexXY) d(opmH) d(pscC), PAO750 PAO397</td><td>>128</td></tr>
<tr><th>Species</th><th>Strain</th><th>Polymyxin B</th><th>Teico-planin</th><th>Genta-micin</th><th>CBD</th></tr>
<tr><td>Acinetobacter baumannii[1]</td><td>ATCC 19606 parent strain</td><td>0.25-0.5</td><td>>128</td><td>32-64</td><td>>128</td></tr>
<tr><td>Acinetobacter baumannii[1]</td><td>AL1851, lpxA mutant; Lipid A deficient</td><td>128->128</td><td>0.125-0.5</td><td>0.5-2</td><td>0.125-0.25</td></tr>
</table>

[1]MIC assays at UQ (n = 4)

Supplementary Table 10. Broth microdilution MIC (μg mL^{-1}) synergy assay of CBD in combination with colistin, polymyxin B and polymyxin B nonapeptide against Gram-negative bacteria. Data is n=2 with all data points presented.

				**CBD MIC (μg mL^{-1}) with Colistin at conc (μg mL^{-1}) of**				Colistin MIC (μg mL^{-1})
Antibiotic	Species	Strain/ Description	CBD MIC (μg mL^{-1})	**0.5**	**0.25**	**0.125**	**0.06**	
Colistin	*Escherichia coli*	ATCC 25922 FDA control	64		1	4	>64	0.5
			>64		0.5	8	>64	0.5
	Klebsiella pneumoniae	ATCC 700603 ESBL	>64		4	>64	>64	0.5
			>64		>64	>64	>64	0.5
	Acinetobacter baumannii	ATCC 19606 Type strain	>64	1	1	16	8	1
			>64	8	>64	>64	>64	1
	Pseudomonas aeruginosa	ATCC 27853 QC strain	>64		>64	>64	>64	0.5

The grey/yellow shaded values indicate synergy, where synergy is defined by FICI value of ≤0.5.
Where no value is given, CDB concentration is irrelevant as the combination is at the MIC concentration of colistin.

**CBD with Colistin FICI values**						
Antibiotic	Species	Strain/ Description	**0.5**	**0.25**	**0.125**	**0.06**
Colistin	*Escherichia coli*	ATCC 25922 FDA control		0.516	0.313	
				0.508	0.375	
	Klebsiella pneumoniae	ATCC 700603 ESBL		0.563		
	Acinetobacter baumannii	ATCC 19606 Type strain	0.516	0.266	0.375	0.185
			0.625			
	Pseudomonas aeruginosa	ATCC 27853 QC strain				

FICI scores were calculated using an MIC of 64 μg mL^{-1} for cannabidiol.
The shaded values indicate synergy, where synergy is defined by FICI value of ≤0.5.
Where an FICI value is not displayed one could not be calculated as there was no relevant inhibitory activity to record, i.e. either CBD inactive >64 μg mL^{-1} or concentration was equal to the MIC of colistin.

				**CBD MIC (μg mL^{-1}) with Polymyxin B at conc (μg mL^{-1}) of**				Polymyxin B MIC (μg mL^{-1})
Antibiotic	Species	Strain/ Description	CBD MIC (μg mL^{-1})	**0.5**	**0.25**	**0.125**	**0.06**	
Polymyxin B	*Escherichia coli*	ATCC 25922 FDA control	64			>64	>64	0.25
			>64		32	16	>64	0.5
	Klebsiella pneumoniae	ATCC 700603 ESBL	>64	4	>64	>64	>64	1
			>64	4	>64	>64	>64	1
	Acinetobacter baumannii	ATCC 19606 Type strain	>64	1	4	>64	>64	1
			>64	>64	>64	>64	>64	1
	Pseudomonas aeruginosa	ATCC 27853 QC strain	>64		>64	>64	>64	0.5
			>64		>64	>64	>64	0.5

The grey/yellow shaded values indicate synergy, where synergy is defined by FICI value of ≤0.5.
Where no value is given, CDB concentration is irrelevant as the combination is at the MIC concentration of polymyxin B.

**CBD with Polymyxin B FICI values**						
Antibiotic	Species	Strain/Description	**0.5**	**0.25**	**0.125**	**0.06**
Polymyxin B	*Escherichia coli*	ATCC 25922 FDA control		1.063		
				1.000	0.5	
	Klebsiella pneumoniae	ATCC 700603 ESBL	0.563			
			0.563			
	Acinetobacter baumannii	ATCC 19606 Type strain	0.516	0.313		
	Pseudomonas aeruginosa	ATCC 27853 QC strain				

FICI scores were calculated using an MIC of 64 μg mL^{-1} for cannabidiol.
The shaded values indicate synergy, where synergy is defined by FICI value of ≤0.5.
Where an FICI value is not displayed one could not be calculated as there was no relevant inhibitory activity to record, i.e. either CBD inactive >64 μg mL^{-1} or concentration was equal to the MIC of polymyxin B.

Antibiotic	Species	Strain/ Description	CBD MIC (µg mL^{-1})	CBD MIC (µg mL^{-1}) with PMBN at conc (µg mL^{-1}) of						PMBN MIC (µg mL^{-1})
				**32**	**16**	**8**	**4**	**2**	**1**	
PMBN	*Escherichia coli*	ATCC 25922 FDA control	>256	64	64	64	64	64	64	>32
			>256	64	>256	>256	>256	64	64	>32
			>256	64	64	64	64	>256	>256	>32
			>256	64	64	>256	>256	>256	>256	>32
	Klebsiella pneumoniae	ATCC 700603 ESBL	>256	64	>256	>256	>256	>256	>256	>32
			>256	32	32	32	64	>256	>256	>32
			>256	>256	>256	>256	>256	>256	>256	>32
			>256	>256	>256	>256	>256	>256	>256	>32
	Acinetobacter baumannii	ATCC 19606 Type strain	>256	32	32	32	32	32	32	>32
			>256	32	32	32	32	32	32	>32
			>256	32	32	32	32	64	>256	>32
			>256	32	32	32	32	64	64	>32
	Pseudomonas aeruginosa	ATCC 27853 QC strain	>256	32	64	64	64	64	64	>32
			>256	32	32	32	32	64	64	>32
			>256	16	32	64	32	32	>256	>32
			>256	16	32	64	32	32	>256	>32

The grey/yellow shaded values indicate synergy, where synergy is defined by FICI value of ≤0.5.
Where no value is given, CDB concentration is irrelevant as the combination is at the MIC concentration of polymyxin B.

CBD with PMBN FICI values								
Antibiotic	Species	Strain/Description	**32**	**16**	**8**	**4**	**2**	**1**
PMBN	*Escherichia coli*	ATCC 25922 FDA control	1.25	0.75	0.5	0.375	0.3125	0.281
			1.25	1.5	1.25	1.125	0.3125	0.281
			1.25	0.75	0.5	0.375	1.062	1.031
			1.25	0.75	1.25	1.125	1.062	1.031
	Klebsiella pneumoniae	ATCC 700603 ESBL	1.25	1.5	1.25	1.125	1.062	1.031
			1.125	0.625	0.375	0.375	1.062	1.031
			2	1.5	1.25	1.125	1.062	1.031
			2	1.5	1.25	1.125	1.062	1.031
	Acinetobacter baumannii	ATCC 19606 Type strain	1.125	0.625	0.375	0.25	0.188	0.156
			1.125	0.625	0.375	0.25	0.188	0.156
			1.125	0.625	0.375	0.25	0.312	1.031
			1.125	0.625	0.375	0.25	0.312	0.281
	Pseudomonas aeruginosa	ATCC 27853 QC strain	1.125	0.75	0.5	0.375	0.312	0.281
			1.125	0.625	0.375	0.25	0.312	0.281
			1.062	0.625	0.5	0.25	0.187	1.031
			1.062	0.625	0.5	0.25	0.187	1.031

FICI scores were calculated using an MIC of 64 µg mL^{-1} for cannabidiol.
The shaded values indicate synergy, where synergy is defined by FICI value of ≤0.5.
Where an FICI value is not displayed one could not be calculated as there was no relevant inhibitory activity to record, i.e. either CBD inactive >64 µg mL^{-1} or concentration was equal to the MIC of polymyxin B.

Supplementary Table 11. Frequency resistance

Titer of 18 h stationary phase culture of *Staphylococcus aureus* (MRSA ATCC 43300)

Dilution Tube	Cfu per plate		Average cfu per plate	Dilution factor	Average cfu mL^{-1}
10^{-6}	1728	2368	2048	10^{7}	2.05×10^{10}
10^{-7}	832	728	780	10^{8}	7.80×10^{10}
10^{-8}	332	264	298	10^{9}	2.98×10^{11}
					**1.32×10^{11}**

Resistance Determination

CBD conc. in plate (Fold MIC)	**Colony Counts on Plate** (MRSA dilution tube/volume applied)				Average cfu per plate	Resistance frequency
	**Neat** in 100µL		**10^{-1}** in 100µL			
16×	0	0	0	0	0	-
8×	0	0	0	0	0	-
4×	0	0	0	0	0	-
2×	5	0	0	0	5	**3.78×10^{-10}**

Supplementary Table 12. Haemolysis Data

**Compound Name**	**ARCBS whole blood**			
	**HC_{10} (µg mL^{-1})**		**HC_{50} (µg mL^{-1})**	
Melittin	3.42	3.63	5.93	5.71
Cannabidiol	>256<br>>256<br>>256	>256<br>>256<br>>256	>256<br>>256<br>>256	>256<br>>256<br>>256

ARCBS, Australian Red Cross Blood Service.
HC_{10}/HC_{50}, concentration required to induce 10% and 50% haemolysis.

Supplementary Table 13. Cytotoxicity Data

**Compound Name**	**HEK-293 (ATCC CRL-1573)**	
	**CC_{50} (µg mL^{-1})**	
Tamoxifen	15.5	13.3
Cannabidiol	231<br>244<br>224	247<br>251<br>194

CC_{50}, concentration required to induce 50% cell cytotoxicity/growth inhibition.

Supplementary Table 14. Topical CBD formulation compositions.

**Formulation Number**	**1**	**2**	**3**	**4**	**5**	**6**	**7**	**8**	**9**	**10**	**11**	**12**
**Ingredients (%w/w)**												
Dow Q7-9180 Silicone Fluid 0.65 cst	0	0	0	92	44.5	13.3	3.5	57.3	42	47	67	33.5
Dow Q7-9120 Silicone Fluid 12500 cst	0	0	0	1	0	2.0	2.5	0	0	0	0	0
Dow 9045 Silicone Elastomer Blend	0	0	0	0	25	69.7	79.0	18.3	0	0	0	0
Dow Corning BY 11-030	0	0	0	0	0	0	0	0	0	0	17	15
Arlamol PS15E	0	14.1	0	2	8	5.0	5.3	4.6	3	10	3	0
Dow Corning 9041 Elastomer Blend	0	0	0	0	0	0	0	0	25	0	0	0
Compritol 888 ATO	0	0	0	0	10	0	0	0	10	0	0	0
Petrolatum	80	0	0	0	2.5	0	0	0	0	0	0	0
Castorwax	0	0	0	0	0	0	0	7.6	0	0	0	0
Isopropyl Alcohol	0	3.4	0	0	0	0	0	2.3	0	3	2	0
Isopropyl Myristate	0	0	0	0	0	0	0	0	5	0	0	0
Plural diisostearique	0	0	0	0	0	0	0	0	2	0	0	0
Monosteol (PG Stearate)	0	0	0	0	0	0	0	0	3	0	0	0
Transcutol	0	62.5	0	0	0	0	0	0	0	20	0	30
PEG 400	0	0	50	0	0	0	0	0	0	0	0	0
PEG 4000	0	0	30	0	0	0	0	0	0	0	0	0
Water	0	0	0	0	0	0	0	0	0	0	1	1.5
**Cannabidiol**	**20**	**20**	**20**	**5**	**10**	**10**	**10**	10	10	**20**	**10**	**20**
