# Supplementary material for: The antimicrobial potential of cannabidiol
Source: Commun Biol. 2021 Jan 19;4:7. doi: 10.1038/s42003-020-01530-y (PMC7815910; doi:10.1038/s42003-020-01530-y)
Supplement: Supplementary file 4 — Reporting Summary [file 42003_2020_1530_MOESM4_ESM.pdf]

nature research

Corresponding author(s): Mark Blaskovich

Last updated by author(s): Nov 20, 2020

Reporting Summary

Nature Research wishes to improve the reproducibility of the work that we publish. This form provides structure for consistency and transparency in reporting. For further information on Nature Research policies, see our Editorial Policies and the Editorial Policy Checklist.

Statistics

For all statistical analyses, confirm that the following items are present in the figure legend, table legend, main text, or Methods section.

n/a	Confirmed	
☐	☒	The exact sample size (n) for each experimental group/condition, given as a discrete number and unit of measurement
☐	☒	A statement on whether measurements were taken from distinct samples or whether the same sample was measured repeatedly
☐	☒	The statistical test(s) used AND whether they are one- or two-sided<br>*Only common tests should be described solely by name; describe more complex techniques in the Methods section.*
☐	☒	A description of all covariates tested
☐	☒	A description of any assumptions or corrections, such as tests of normality and adjustment for multiple comparisons
☐	☒	A full description of the statistical parameters including central tendency (e.g. means) or other basic estimates (e.g. regression coefficient) AND variation (e.g. standard deviation) or associated estimates of uncertainty (e.g. confidence intervals)
☐	☒	For null hypothesis testing, the test statistic (e.g. F, t, r) with confidence intervals, effect sizes, degrees of freedom and P value noted<br>*Give P values as exact values whenever suitable.*
☒	☐	For Bayesian analysis, information on the choice of priors and Markov chain Monte Carlo settings
☒	☐	For hierarchical and complex designs, identification of the appropriate level for tests and full reporting of outcomes
☒	☐	Estimates of effect sizes (e.g. Cohen's d, Pearson's r), indicating how they were calculated

Our web collection on statistics for biologists contains articles on many of the points above.

Software and code

Policy information about availability of computer code

Data collection	No data collection code software was used in this study.
Data analysis	Prism 8 software, Microsoft Excel, Fiji (Image J), Lumina II system software (Perkin-Elmer)

For manuscripts utilizing custom algorithms or software that are central to the research but not yet described in published literature, software must be made available to editors and reviewers. We strongly encourage code deposition in a community repository (e.g. GitHub). See the Nature Research guidelines for submitting code & software for further information.

Data

Policy information about availability of data

All manuscripts must include a data availability statement. This statement should provide the following information, where applicable:

- Accession codes, unique identifiers, or web links for publicly available datasets
- A list of figures that have associated raw data
- A description of any restrictions on data availability

The data that support the findings of this study are either available within the paper [and its supplementary information files] or available from the corresponding author upon reasonable request.

Field-specific reporting

Please select the one below that is the best fit for your research. If you are not sure, read the appropriate sections before making your selection.

☒ Life sciences ☐ Behavioural & social sciences ☐ Ecological, evolutionary & environmental sciences

For a reference copy of the document with all sections, see nature.com/documents/nr-reporting-summary-flat.pdf

Life sciences study design

All studies must disclose on these points even when the disclosure is negative.

Sample size	For in vivo efficacy studies, group size is kept as small as possible to yield quality data and is dictated by industry standards and established practice for the relevant models, not statistical analysis. All in vivo study protocols used in this work stated, "animal numbers are as low as possible to obtain significant difference between groups, and no alternative methods are available for the study". This was accepted by the Ethics committees who approved each study.
Data exclusions	Data was not excluded.
Replication	All in vitro studies studies conducted at The University of Queensland and Monash University were repeated at minimum of 2 technical replicates, with MIC determinations consisting of at least 2 biological replicates on independent days with independent prepared samples and reagents, with 2 technical replicates on each day (n >=4). For MIC studies conducted at Micromyx, for some studies only n=1 was performed, but most studies used n=3 based on three independent starting inocula. For ex vivo studies measuring bacteria reduction on pig skin conducted at Extherid, at least 2 biological replicates on independent days with at least 2 technical replicates each day were conducted. For in vivo studies technical replicates (i.e. multiple animals) were used in the one assay. Biological replication for in vivo studies was not conducted in order to conserve animal usage.
Randomization	Randomization was used in the selection of animals to be used in each of the in vivo studies from cohorts specifically bred for research purposes. Randomization is not relevant to the rest of our studies as no population, clinical data or field studies where randomization is required was carried out. All work was direct experimental design and all output used in each set of analysis.
Blinding	All studies were carried out in a blind fashion.

Reporting for specific materials, systems and methods

We require information from authors about some types of materials, experimental systems and methods used in many studies. Here, indicate whether each material, system or method listed is relevant to your study. If you are not sure if a list item applies to your research, read the appropriate section before selecting a response.

Materials & experimental systems

n/a	Involved in the study	
☒	☐	Antibodies
☐	☒	Eukaryotic cell lines
☒	☐	Palaeontology and archaeology
☐	☒	Animals and other organisms
☐	☒	Human research participants
☒	☐	Clinical data
☒	☐	Dual use research of concern

Methods

n/a	Involved in the study	
☒	☐	ChIP-seq
☒	☐	Flow cytometry
☒	☐	MRI-based neuroimaging

Eukaryotic cell lines

Policy information about cell lines

Cell line source(s)	The eukaryotic cell line used in this study was sourced from the American Type Culture Collection (ATCC): HEK-293 human embryonic kidney cells (ATCC CRL-1573).
Authentication	Cell lines were authenticated by ATCC standard STR profiling guidelines.
Mycoplasma contamination	Cell lines were tested for Mycoplasma contamination using the MycoAlert Mycoplasma Detection Kit (Cat #LT07-118).
Commonly misidentified lines (See ICLAC register)	No commonly misidentified cell lines were used in the study.

Animals and other organisms

Policy information about studies involving animals; ARRIVE guidelines recommended for reporting animal research

Laboratory animals	Thigh infection model: Seven-week-old female outbred CD1 mice (UQBR-AIBN, UQ). Animals were housed 5 per cage and maintained in the infectious room of UQBR with a 12 hour day 12 hour night cycle and controlled temperature, each cage provided with sheets of tissue paper, wood chips and/or other environmental enrichment toys (carton cylinders, etc).<br>Bioluminescence model: Seven- to ten-week-old female CD1 mice (Charles River laboratories, no additional information provided by service provider).
Wild animals	No wild animals were used in this study.
Field-collected samples	No field collected samples were used in this study.
Ethics oversight	The thigh infection studies were conducted at the University of Queensland and approved by the Molecular Biosciences Animal Ethics Committee, approval number IMB_464_17 2018. The mouse bioluminescent skin infection model was performed by Charles River Laboratories, Portishead UK under UK Home Office Licenses and with local ethical committee clearance.

Note that full information on the approval of the study protocol must also be provided in the manuscript.

Human research participants

Policy information about studies involving human research participants

Population characteristics	All sourced blood for haemolysis studies was donated by the Australian Red Cross Blood Service and no patient information is supplied to the researchers of this project as per the agreement with the Australian Red Cross and The University of Queensland Human Ethics Approval, therefore population characteristics are unknown.
Recruitment	All sourced blood for haemolysis studies was donated by the Australian Red Cross Blood Service and no recruitment information is supplied to the researchers of this project as per the agreement with the Australian Red Cross and The University of Queensland Human Ethics Approval, therefore recruitment information is unknown.
Ethics oversight	The use of human blood (sourced from the Australian Red Cross Blood Service) for haemolysis assays was approved by the University of Queensland Institutional Human Research Ethics Committee, Approval Number 2014000031.

Note that full information on the approval of the study protocol must also be provided in the manuscript.
