# Supplementary material for: The antimicrobial potential of cannabidiol
Source: Commun Biol. 2021 Jan 19;4:7. doi: 10.1038/s42003-020-01530-y (PMC7815910; doi:10.1038/s42003-020-01530-y)
Supplement: Supplementary file 5 — Description of Additional Supplementary Files [file 42003_2020_1530_MOESM5_ESM.pdf]

Description of Additional Supplementary Files

File name: Supplementary Data 1

Description: Source data for figures.
